# Autoimmunity promotes chronic lymphocytic leukemia progression in an indolent disease model

**DOI:** 10.1038/s41598-025-86876-1

**Published:** 2025-02-03

**Authors:** Lisa Pfeuffer, Viola Siegert, Riccardo Trozzo, Katja Steiger, Roland Rad, Jürgen Ruland, Maike Buchner

**Affiliations:** 1 Institute of Clinical Chemistry and Pathobiochemistry, Technical University of Munich, TUM School of Medicine and Health, Ismaninger Str. 22, 81675 Munich, Germany; 2 TranslaTUM, Center for Translational Cancer Research, Technical University of Munich, Munich, Germany; 3 Institute of Molecular Oncology and Functional Genomics, TUM School of Medicine and Health, Technical University of Munich, 81675 Munich, Germany; 4 Institute of Pathology, Technical University of Munich, Munich, Germany; 5 German Cancer Consortium (DKTK), partnership between German Cancer Research Center (DKFZ) and TUM University Hospital, Munich, Germany; 6 German Center for Infection Research (DZIF), Munich partner site, Munich, Germany

**Keywords:** Cancer models, Chronic lymphocytic leukaemia

## Abstract

Chronic lymphocytic leukemia (CLL) is a heterogeneous B cell malignancy characterized by the accumulation of functionally incompetent B lymphocytes. Despite the availability of highly effective treatments, CLL remains incurable, and the factors contributing to disease progression are not fully understood. Autoimmune complications frequently arise in CLL patients and are associated with poor clinical prognosis. This study investigates the connection between plasma cell-mediated autoimmunity and CLL progression using a mouse model that expresses an active Receptor Activator of NF-κB (RANK) in B cells (RK mice), where autoimmune manifestations coexist with CLL. Transcriptional profiling of RANK-driven leukemic cells revealed a more indolent form of CLL compared to the classical TCL1 model. The discovery of near-identical CDR3 regions in both plasma and CLL cells of RK mice suggests a shared progenitor and antigen driving both conditions. Deletion of Blimp-1, which prevents plasma cell differentiation, initially enhanced B1/CLL formation in young mice but nearly halted CLL progression, highlighting the significant influence of autoimmune complications on disease outcomes. This research underscores the intertwined nature of autoimmunity and CLL, suggesting that targeting inflammatory pathways could offer therapeutic potential for managing both conditions.

**Supplementary Information:**

The online version contains supplementary material available at 10.1038/s41598-025-86876-1.

## Introduction

B cells are essential to adaptive immunity through their role in antibody production, but they can also contribute to the development of autoimmune diseases and B cell lymphomas. During B cell development, recombination processes and somatic hypermutations generate up to 10^18^unique B cell receptor (BCR) structures, enabling broad antigen recognition. However, these same mechanisms increase the risk of B cells producing autoreactive BCRs and undergoing malignant transformation^1^. To counteract these risks, B cells have evolved mechanisms to eliminate autoreactive cells and prevent transformation. Despite these safeguards, escape mechanisms can arise, with evidence suggesting overlap between pathways driving both autoimmunity and lymphoma^2^. For example, B cells producing harmful autoantibodies often harbor lymphoma-associated mutations affecting NF-κB signaling, the cell cycle, and antibody production^3^. This indicates that mutations arising in B cell precursors may predispose individuals to both autoimmune diseases and lymphoma. Moreover, B cell-driven autoimmune diseases, such as systemic lupus erythematosus (SLE), rheumatoid arthritis, and Sjögren’s syndrome, significantly increase the risk of developing diffuse large B cell lymphoma (DLBCL), a common subtype of non-Hodgkin lymphoma^4–6^. These findings suggest a specific overlap in the pathogenesis of autoimmunity and B cell lymphomagenesis. Further research is needed to elucidate the co-evolution of B cell-mediated autoimmunity and malignant transformation, and how autoimmunity may drive lymphomagenesis.

Chronic Lymphocytic Leukemia (CLL) is a highly heterogeneous disease, ranging from indolent to aggressive forms. B cell receptor (BCR) signaling plays a central role in determining the clinical course. CLL is classified based on the mutational status of the immunoglobulin heavy-chain variable region gene (IGHV) into two subtypes: mutated IGHV (M-CLL) and unmutated IGHV (U-CLL). U-CLL, which typically exhibits more aggressive progression and poorer outcomes^7,8^, is characterized by BCRs that frequently recognize a broad range of autoantigens. In contrast, M-CLL BCRs bind antigens with higher specificity and affinity. Reconstruction of mutated, non-autoreactive antibody sequences back to their unmutated forms has revealed that the germline BCRs are polyreactive and autoreactive, indicating that somatic hypermutation alters the autoreactivity of the BCR. This suggests that both CLL subtypes originate from self-reactive B cell precursors, and the degree of BCR polyreactivity—and consequently autoantigen recognition—correlates with the clinical course of the disease^9^. Furthermore, BCR repertoire analysis has revealed a restricted use of IGHV genes, often paired with specific IGHD and IGHJ segments, resulting in near-identical sequences in the variable complementarity-determining region 3 (CDR3). These stereotyped receptors support the idea that CLL BCRs often recognize a limited set of (auto)antigens^10^. Stereotyped BCRs are more prevalent in U-CLL and are strongly associated with poor prognosis and a higher risk of transformation to Richter syndrome^11,12^. For example, subset 2 CLL, characterized by IGHV3-21 usage and the distinctive VH CDR3 motif DANGMDV, is linked to aggressive disease progression, poor clinical outcomes, and frequent del11q22-q23 abnormalities. Similarly, subset 8 CLL, defined by IGHV4-39 usage, is associated with an exceptionally high risk of Richter transformation, further highlighting the role of antigen-driven stimulation in driving disease progression. These findings underscore the strong association between stereotyped BCR subsets and adverse clinical outcomes, although rare subsets such as subset 4 may exhibit a more indolent course. Finally, aberrant expression of ZAP-70, a tyrosine kinase functionally similar to SYK and operating downstream of the BCR, is associated with a poorer prognosis, further underscoring the relationship between BCR signaling, disease progression, and prognosis^13,14^.

In addition to the frequently autoreactive malignant B cells, CLL patients often develop autoimmune complications, affecting 7–25% of cases^15–17^. The most common autoimmune disorders associated with CLL are autoimmune cytopenias, such as autoimmune hemolytic anemia (AIHA) and immune thrombocytopenia (ITP). These conditions are caused by autoantibodies produced by non-malignant B cell clones that target membrane antigens on red blood cells (in AIHA) or platelets (in ITP). These autoantibodies lead to the destruction of these blood cells, contributing to anemia and thrombocytopenia in affected patients. The pathophysiology of autoimmune disorders in CLL likely involves a range of humoral and cellular immune dysfunctions, though this is not fully understood. However, several studies have established a significant association between autoimmune disorders and unfavorable prognostic markers in CLL, such as high lymphocyte count, elevated β2-microglobulin levels, and increased expression of CD38 and ZAP-70^18,19^. These autoimmune complications are also more frequent in U-CLL patients^20^. Autoimmune complications also impact disease management, often necessitating the use of glucocorticoids or rituximab to eliminate both malignant and autoreactive B cells^21,22^. Additionally, therapies targeting B cell receptor signaling pathways, such as the Bruton tyrosine kinase (BTK) inhibitor ibrutinib or the phosphoinositide 3-kinase (PI3K) inhibitor idelalisib, have shown efficacy in not only controlling CLL but also addressing associated autoimmune manifestations^23–25^. These therapies not only manage the direct malignant features of CLL but also mitigate the autoimmune manifestations that often accompany the disease, suggesting a complex interplay between CLL progression and immune dysfunction. However, despite these advances, the potential role of autoimmune complications in driving CLL progression has not been thoroughly investigated.

Our previous work demonstrated that B cell-specific expression of a mutated Receptor Activator of NF-κB (RANK^K240^), identified in human diffuse large B cell lymphoma (DLBCL) patients^26^, triggers autoimmune disease in mice (RK mice). This disease is characterized by hyperactive B cells, anti-nuclear antibodies, and glomerulonephritis. In aged mice, active RANK expression further leads to the malignant transformation of B1 B cells, mimicking human and murine CLL^27^. However, it remains unclear whether the autoimmune phenotype contributes to CLL progression or worsens prognosis. To address this, we have now studied the co-occurrence of autoimmunity and CLL, as well as the role of an inflammatory environment in CLL progression in our RK-driven mouse model. Our findings underscore the critical role of plasma cell-mediated autoimmunity in promoting CLL development, likely through the modulation of a pro-inflammatory environment.

## Results

### Autoimmunity and CLL development co-develop in RANKK240E transgenic mice

**Fig. 1:**
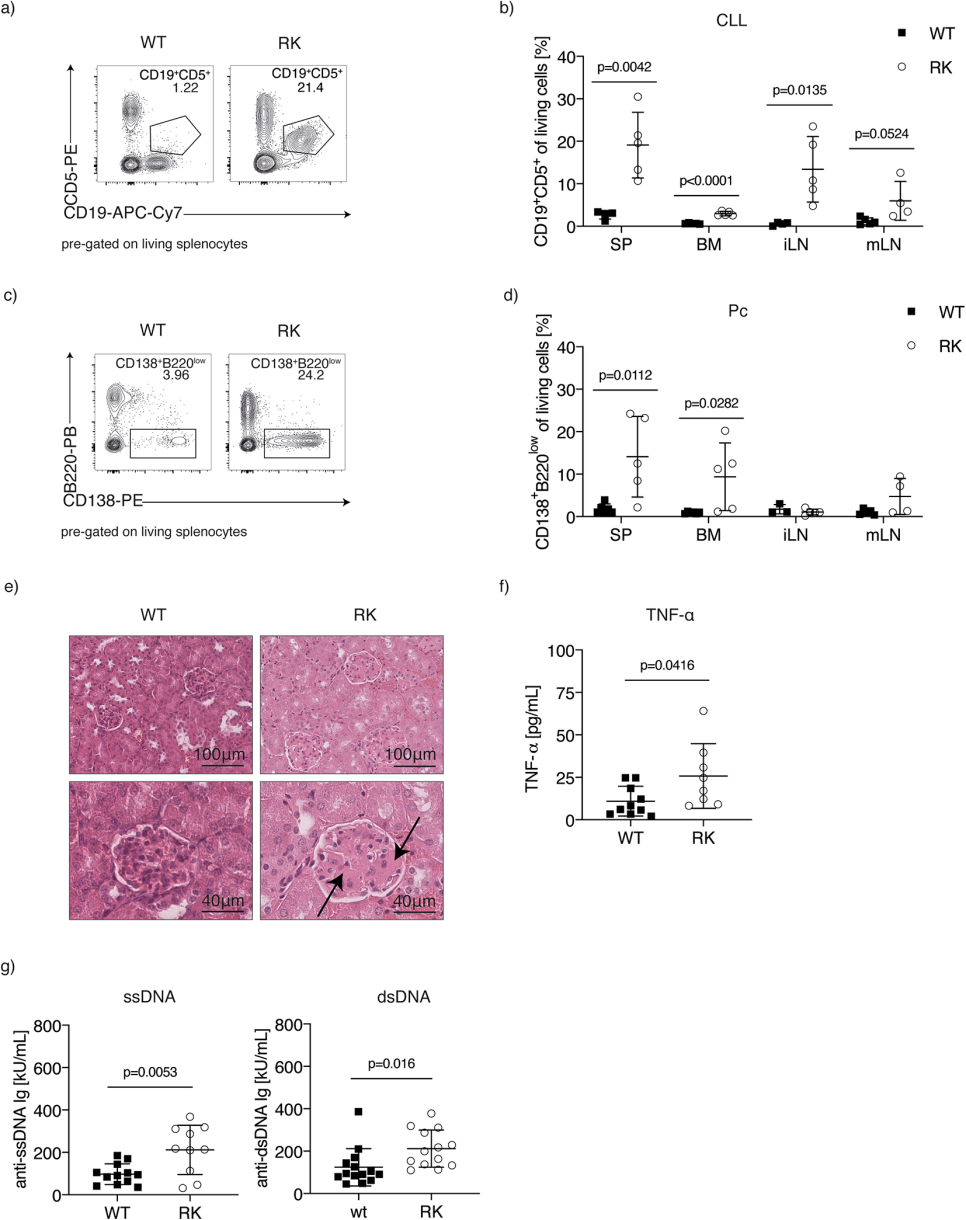
Coexistence of CLL- and autoimmunity-like disease in strict SPF housed RANK^K240E^transgenic mice. **a** Representative flow cytometric analysis of CD19 and CD5 expression on viable splenocytes from aged RK and WT mice. Gating and percentage of CD19^+^CD5^+^ cells are indicated in the respective graphs. **b** Percentages of CD19^+^CD5^+^ cells of living cells isolated from aged RK and WT mice (*n* = 4–5 per organ). **c** Representative flow cytometric analysis of CD138 and B220 expression on viable splenocytes from aged RK and WT mice. Gating and percentage of CD138^+^B220^low^ cells are indicated in the respective graphs. **d** Percentages of CD138^+^B220^low^ plasma cells (Pc) of living cells isolated from aged RK and WT mice which were also analyzed in b (*n* = 4–5 per organ and genotype). **e** Representative images of HE staining from kidneys of aged WT and RK mice (40x magnification; overview: scale bar = 100 μm, detailed image: scale bar = 40 μm). **f** Quantification of TNF-α levels in plasma samples from aged RK (*n* = 8) and WT mice (*n* = 10) determined by flow cytometry-based multiplex immunoassay. Pooled data from two independent experiments. **g** Plasma levels of total anti-ssDNA and anti-dsRNA immunoglobulins in aged RK (*n* = 10–13) and WT (*n* = 12–14) mice using the Alpha Diagnostic International Autoimmunity ELISA kits. Data were pooled from two independent experiments. Statistical analysis was performed using unpaired Student’s *t* test for comparison of two groups. *P* values are indicated in respective graphs. All data are presented as mean ± standard deviation.

To investigate the link between autoimmunity and CLL progression, we first examined the co-evolution of autoimmune disease and CLL in our RANK-driven model. We analyzed aged RK mice for autoimmunity and CLL presence in various lymphoid organs, using plasma cell levels as an indicator of autoimmune activation. Consistent with previous findings, RK mice exhibited significant CLL cell accumulation and increased plasma cell levels in the spleen, bone marrow, and lymph nodes compared to controls^27^(Fig. 1a-d). The enrichment of plasma cells (Pc) in the bone marrow and extramedullary sites highlights the presence of autoimmune inflammation. These findings indicate that the coexistence of CLL and plasma cells, despite their distinct organ-specific distributions, reflects an intricate interplay between malignant and autoimmune processes, with each cell type relying on its respective physiological niche. Notably, both CLL and autoimmune manifestations were less pronounced in our current study compared to our previous findings, where mice housed under lower hygiene levels (non-specific-pathogen-free, non-SPF conditions) exhibited more robust phenotypes of both CLL and autoimmunity^27^.

Additionally, renal pathology was assessed via histology, which confirmed protein accumulation in the glomeruli of terminally ill mice (Fig. 1e), indicating that renal failure may contribute to disease severity in RK mice. We also analyzed several cytokines in the sera of RK and WT mice and found elevated levels of tumor necrosis factor-alpha (TNF-α) in RK mice compared to age-matched WT controls (Fig. 1f). In addition, autoantibody levels in plasma revealed significantly elevated anti-nuclear antibodies (ANAs) binding ssDNA and dsDNA in RK mice compared to age-matched controls (Fig. 1g). However, these autoantibody levels were lower and appeared later in life in RK mice housed under SPF conditions compared to those in less stringent environments^27^, suggesting that immune triggering exacerbates autoimmunity in our model. Although investigating the causes of this shift is intriguing, it is not the primary focus of this study. The observed correlation between reduced autoimmunity and slower CLL progression in RK mice led us to hypothesize that autoimmune mechanisms may play a driving role in CLL development. This is particularly relevant given the well-established association between autoimmunity and poor clinical outcomes in CLL patients^18–20^. Therefore, the co-occurrence of autoimmunity and CLL in RK mice positions them as a valuable model for investigating whether autoimmunity might impact CLL progression.

## Transcriptomic analysis indicates upregulation of inflammatory signatures in RK-derived CLL

**Fig. 2:**
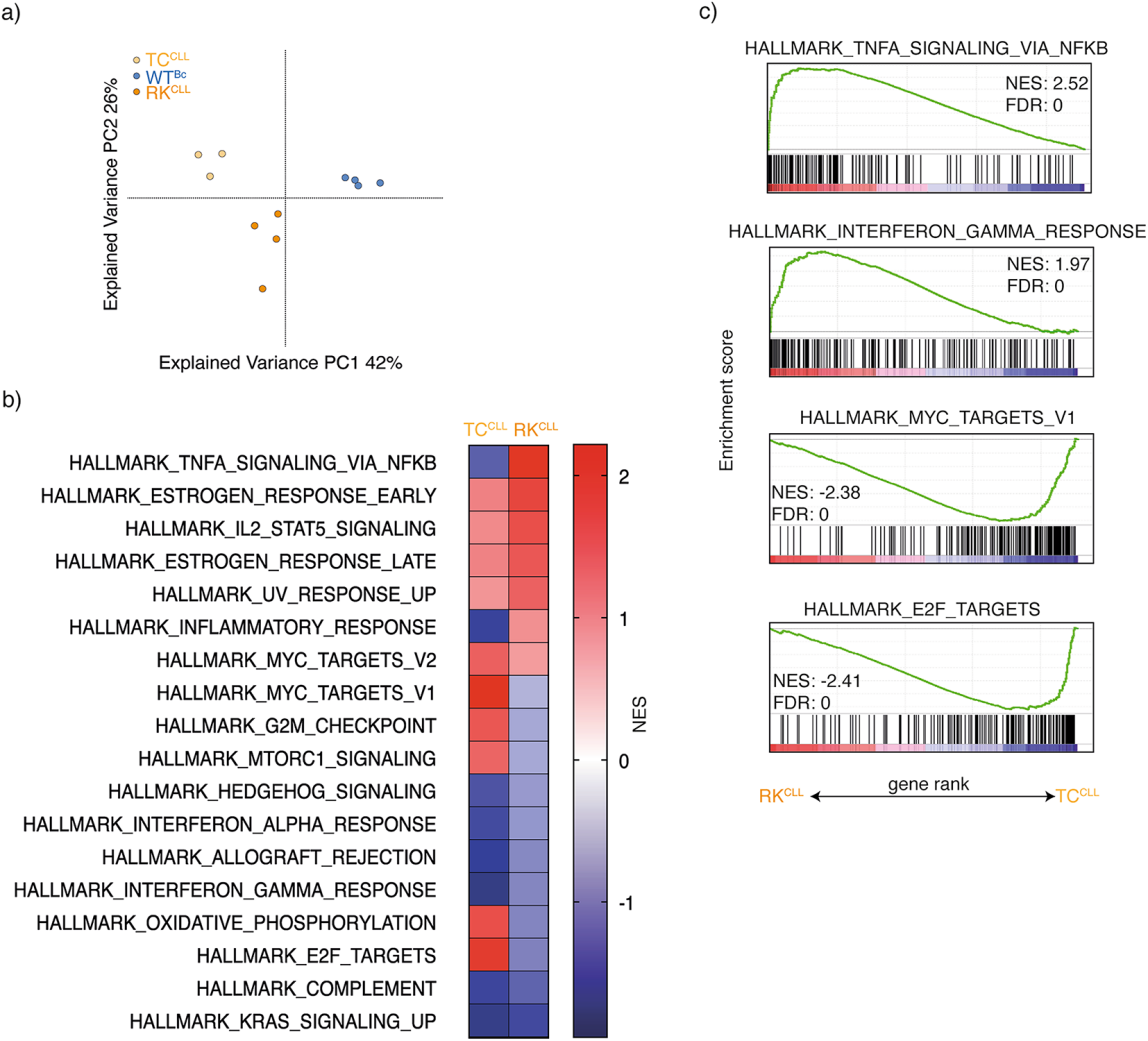
Transcriptomic analysis indicates upregulation of inflammatory signatures in RK-derived compared to TC-derived CLL. **a** Principal component analysis of transcriptome from CD19^+^CD5^+^ CLL cells isolated from diseased TC (TC^CLL^, *n* = 3) and RK mice (RK^CLL^, *n* = 4), along with CD19^+^ B cells from aged-matched WT mice (*n* = 3). **b** Gene set enrichment analysis (GSEA) analysis showing hallmarks enriched in splenic B1a cells isolated either from diseased TC (TC^CLL^, *n* = 3) or RK mice (RK^CLL^, *n* = 4) compared to CD19^+^ B cells from aged-matched WT mice (*n* = 3). Hallmarks and NES (normalized enrichment score) are shown, if false discovery rate (FDR) ≤ 0.05 for TC or RK comparison. **c** Gene set enrichment analysis (GSEA) analysis showing hallmarks enriched in splenic B1a cells isolated from diseased TC mice (TC^CLL^, *n* = 3) compared to aged RK mice (RK^CLL^, *n* = 4). NES, normalized enrichment score; FDR, false discovery rate.

To further explore mechanisms driving disease progression in RK mice, we conducted bulk RNA sequencing and compared the transcriptomes of splenic WT B cells with those of leukemic cells from RK mice and the classical TCL1-driven CLL (TC) model^28^. The TCL1 oncogene induces CLL at an earlier age and leads to more pronounced splenomegaly, whereas RANK^K240E^-driven CLL exhibits a more indolent, chronic accumulation with lower expansion rates. These differences suggest distinct driving mechanisms between the two models. To investigate their transcriptional profiles, CD19^+^CD5^+^ splenocytes were isolated from RK and TC mice and compared to CD19^+^ B cells from age-matched WT mice using bulk RNA sequencing. Principal component analysis revealed that CLL cells from RK and TC mice formed distinct clusters, both clearly separated from CD19^+^ B cells of WT mice, indicating a shared yet distinct transcriptome profile between the CLL cells of these two models (Fig. 2a).

Gene set enrichment analysis (GSEA) of the transcriptional profiles of CLL models versus WT B cells showed several shared and some distinct hallmark pathways (Fig. 2b). Both CLL models upregulated factors commonly associated with CLL, including BCR-related genes such as SYK, BLK, and NFATc1, as well as genes involved in B cell activation and survival (BCL2, MYC, CD80) and T cell interaction/suppression (CTLA4, IL10, CD39). Additionally, common negative regulators, including CD300e, CD22, and several complement factors, were downregulated in both CLL models, consistent with findings in human CLL. A complete list of commonly deregulated genes is provided in Supplementary Table 1, highlighting genes identified in both models as significantly deregulated compared to WT B cells, with an adjusted p-value below 0.05. This analysis demonstrates that the two CLL models, driven by TCL1 or RANK^K240E^, share common features, including an upregulation of anti-apoptotic BCL2 and factors facilitating immune evasion.

We next conducted a direct comparison of the RK and TC CLL transcriptomes by GSEA to highlight the differences between the models. This analysis revealed that “TNFA signaling via NF-κB” and “Interferon Gamma and Inflammatory Response” were the most upregulated hallmarks in RK mice, whereas “MYC Target Genes V1” and “E2F Targets” were highly enriched in TC mice (Fig. 2c, Suppl. Table 2, Suppl. Figure 1). These findings align with the observation that TC-driven disease mirrors aggressive CLL^29^, lacking significant levels of autoantibodies (Suppl. Figure 2a, b), while RK-driven disease appears to mimic a more chronic CLL expansion. This suggests that a pro-inflammatory microenvironment may play a crucial role in driving the progression of indolent CLL in RK mice.

## B cell receptor sequencing reveals clonal expansion and (auto)antigen specificity in CLL and plasma cells

**Fig. 3:**
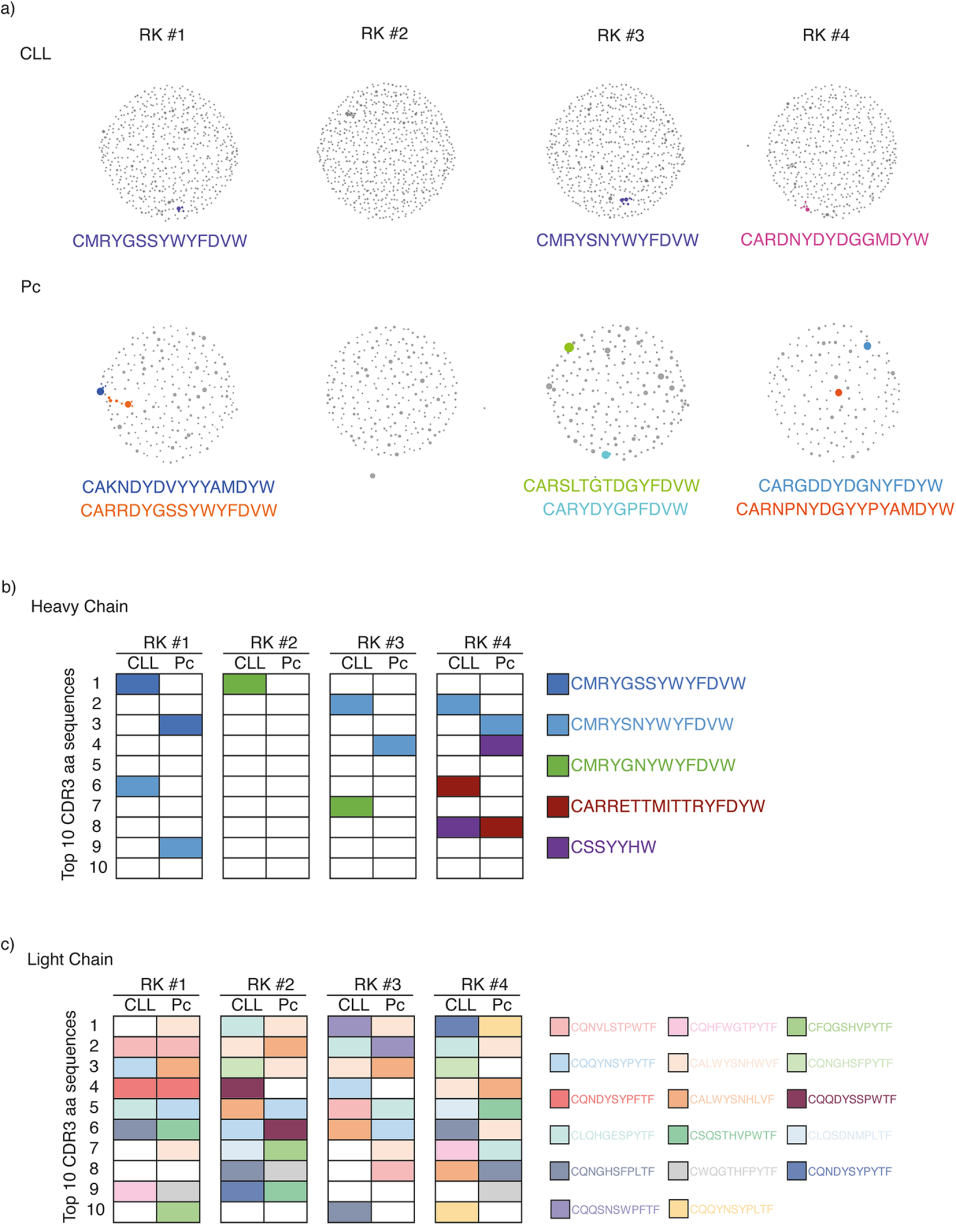
Stereotyped BCRs in CLL and Plasma Cells from RK mice. **a***Igh* clonality analysis from of CD19^+^CD5^+^ B1a/CLL cells and CD138^+^ cells isolated from four different six-month-old RK mice. **b** Scheme of recurrent CDR3 amino acids (aa) sequences within the first ten *Igh* CDR3 regions reconstructed from RNA sequencing of CD19^+^CD5^+^ CLL cells and CD138^+^ plasma cells (Pc) isolated from four different six-month-old RK mice. **c** Scheme of recurrent CDR3 amino acids sequences within the first ten *Igl* and *Igk* CDR3 regions reconstructed from RNA sequencing of CD19^+^CD5^+^ CLL cells and CD138^+^ plasma cells isolated from four different to six-month-old RK mice.

To investigate the expansion within the B cell compartment in our model, we next analyzed the BCR sequences from B1/CLL and plasma cells isolated from six-month-old RK mice by RNA sequencing, prior to onset of disease. At this early disease stage, we expected high BCR diversity and minimal clonal expansion within the individual subsets. Reconstruction of *Igh* sequences revealed early and modest clonal expansion of specific B1/CLL and plasma cell populations at this early time point in three out of four RK mice (Fig. 3a). Notably, we observed recurrent BCR clones across different mice. To explore this further, we compared the top ten CDR3 amino acid sequences of heavy and light chains, both within subsets from a single mouse and across different RK mice, to detect stereotyped receptors. This analysis revealed recurring identical amino acid sequences in both CLL and plasma cells within individual mice, as well as across different RK donor mice (Fig. 3b, c; Suppl. Figure 2c). These findings suggest that both CLL and plasma cells may originate from common autoreactive B cell precursors in RK mice, with a potentially similar (auto)antigen driving the expansion of CLL and autoreactivity.

### **Blimp-1 deletion does not impair CLL formation or viability but promotes initial B1/CLL expansion in RK-BL**^**KO**^**mice**

**Fig. 4:**
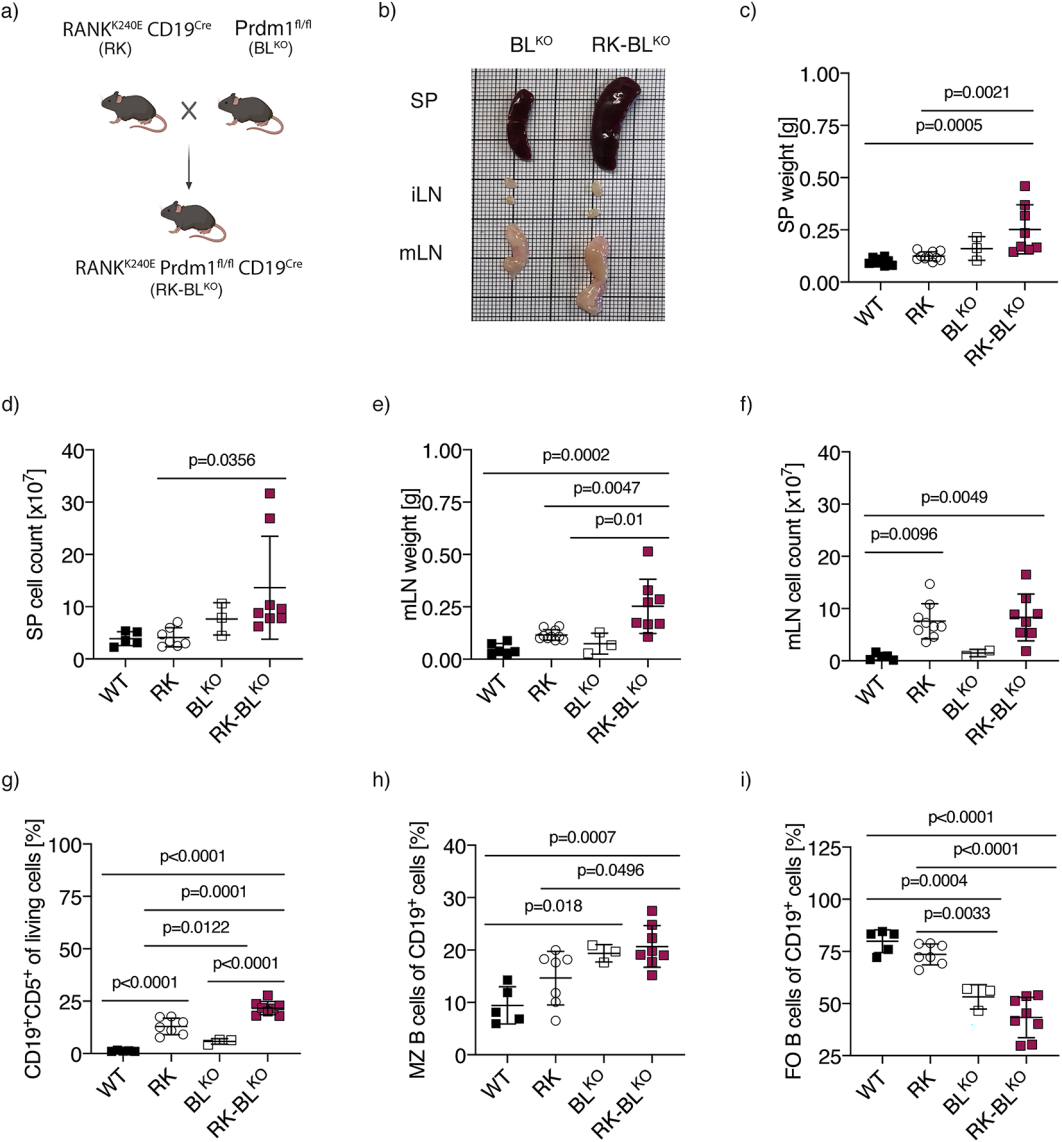
Initial B1/CLL Expansion in RK-BL^KO^mice. **a** Breeding scheme for generating RK-BL mice. Scheme was generated with BioRender.com. **b** Macroscopic appearance of representative spleens (SP), inguinal lymph nodes (ilN) and mesenteric lymph nodes (mLN) from six-month-old RK-BL^KO^ and BL^KO^ mice. **c** Dot plot graph depicts spleen (SP) weight in gram (g) of six-month-old mice with indicated genotypes (*n* = 3–10). **d** Splenic cell count of living cells isolated from six-month-old mice with indicated genotypes (*n* = 3–8). **e** Dot plot graph depicts mesenteric lymph node (mLN) weight in gram (g) of six-month-old mice with indicated genotypes (*n* = 3–10). **f** Cell count of living cells isolated from the mesenteric lymph nodes from five- to six-weeks old mice with indicated genotypes (*n* = 2–9). **g** Percentages of CD19^+^CD5^+^ cells of living splenocytes isolated from six-month-old RK-BL^KO^ mice (*n* = 8) and control mice (*n* = 3–7). **h** Percentages of marginal zone (MZ) B cells (defined by CD21^+^C23^neg^ expression) pre-gated on CD19^+^CD5^neg^ splenocytes isolated from six-month-old RK-BL^KO^ mice (*n* = 8) and age-matched control mice (*n* = 3–7). **i** Percentages of follicular (FO) B cells (defined by CD21^low^CD23^+^ expression) pre-gated on CD19^+^CD5^neg^ splenocytes isolated from six-month-old RK-BL^KO^ mice (*n* = 8) and age-matched control mice (*n* = 3–7). Statistical analysis was performed using one-way ANOVA with Tukey correction for multiple comparison. *P* values are indicated in respective graphs. All data are presented as mean ± standard deviation.

Blimp-1 is a transcription factor essential for plasma cell differentiation, and its loss prevents autoantibody formation^30^. To investigate the impact of autoinflammation on CLL progression, we examined whether depleting Blimp-1 could mitigate autoimmunity in our model. We blocked plasma cell formation by genetically deleting Prdm1, the gene encoding Blimp-1, by crossing them with Prdm1^fl/fl^mice^31^ (BL^KO^) resulting in triple transgenic RANK^K240E^ Prdm1^fl/fl^ CD19^Cre^ (RK-BL^KO^) mice (Fig. 4a). Consistent with previous studies^32^, we confirmed that Blimp-1 is predominantly expressed in plasma cells from RK mice, while it is undetectable in CD19⁺ B cells (similar to BL^KO^ B cells) and expressed at low levels in CLL cells within the same model (Suppl. Figure 3a). Correspondingly, RK-derived leukemic cells and WT-derived CD19^+^ B cells retained expression of Blimp-1 target genes such as *Bcl6*, *Foxo1* and *Pax5* (Suppl. Figure 3b) indicating limited transcriptional activity. Next, we aimed to rule out B cell-intrinsic defects due to B cell-specific Blimp-1 depletion in RK-driven CLL. First, we evaluated normal B cell development and maturation in three- to six-month-old mice. RK-BL^KO^ mice exhibited a mild reduction in B220^+^CD19^+^ cells in the bone marrow compared to WT and BL^KO^ control mice (Suppl. Figure 3c). Further phenotyping revealed an expansion of pro- and pre-B cell populations in RK-BL^KO^ mice compared to controls (Suppl. Figure 3d-f). However, these changes were minor and unlikely to contribute to significant B cell phenotypes in peripheral tissues.

Next, we analyzed B1/CLL expansion in six-month-old RK-BL^KO^ mice. Surprisingly, RK-BL^KO^ mice displayed enhanced splenomegaly compared to WT and RK mice, as indicated by increased spleen weight and splenocyte counts (Fig. 4b-d). This was also observed in the mesenteric lymph nodes (Fig. 4b, e, f). Flow cytometric analysis of the splenic B cell compartment revealed the highest percentages of CD19^+^CD5^+^ cells in RK-BL^KO^ mice compared to WT, RK, and BL^KO^ mice (Fig. 4g). Additionally, we observed increased proportions of CD21^+^ marginal zone (MZ) B cells and reduced proportions of CD23^+^ follicular (FO) B cells in both RK-BL^KO^ and BL^KO^mice, consistent with previous findings^33^ (Fig. 4h-i, Suppl. Figure 4a). These results suggest that the loss of B cell-intrinsic Blimp-1 promotes B1/CLL and MZ expansion, which is further amplified in the presence of active RANK signaling.

To investigate whether Blimp-1 loss affects CLL cell viability, we isolated CD19^+^ B cells including CLL cells from RK and RK-BL^KO^ mice and assessed their survival in vitro. No significant differences in survival were observed in survival and CLL proportion (Suppl. Figure 4b, c). Collectively, these findings suggest that Blimp-1 depletion does not impair CLL development, initial expansion, or survival. Thus, we conclude that Blimp-1 depletion provides a valuable tool to study the role of autoantibodies derived from plasma cells in CLL and autoimmune-driven inflammation.

## Deletion of Blimp-1 prevents autoantibody formation and CLL expansion in aged RK mice

**Fig. 5:**
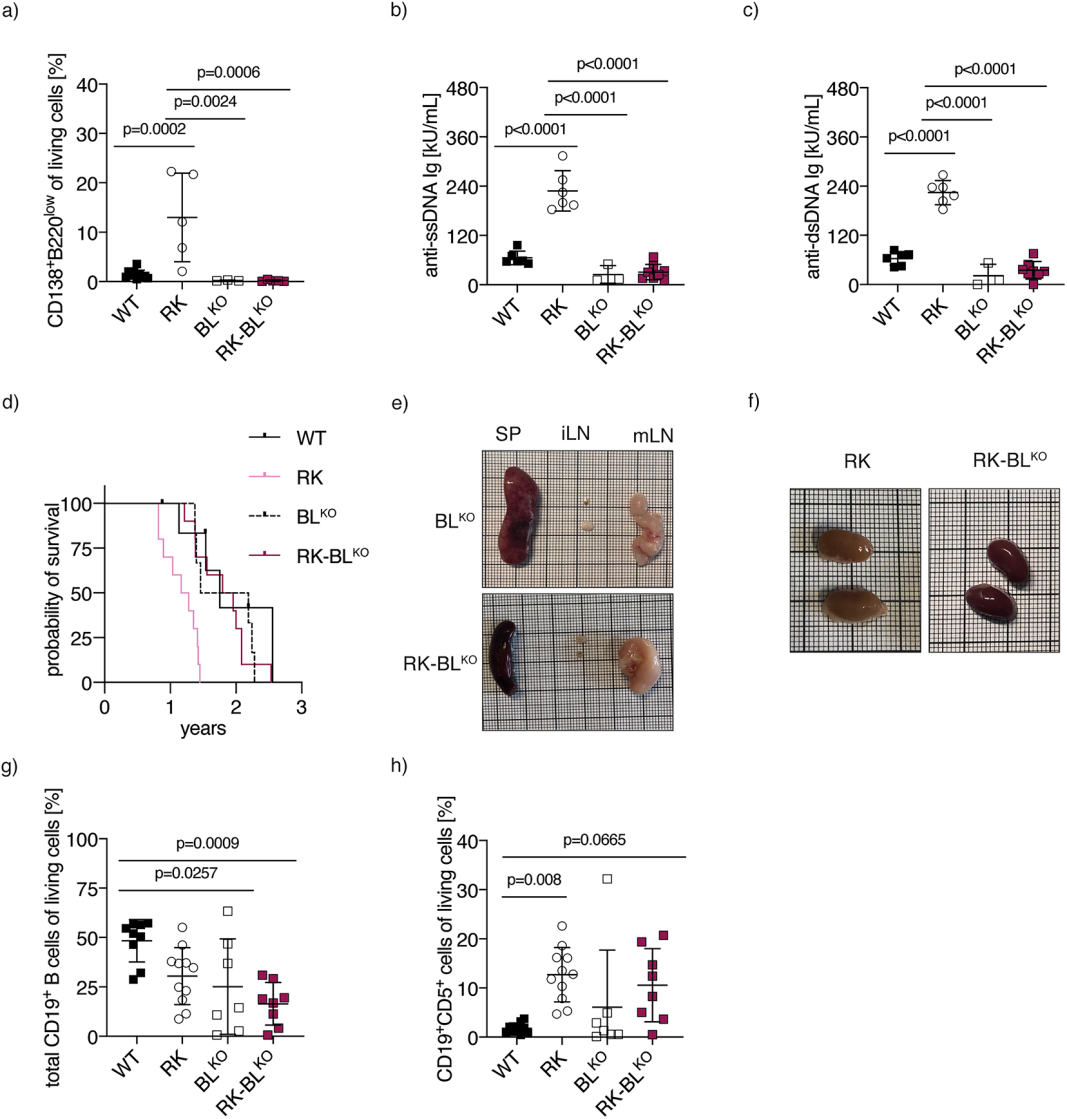
Loss of Blimp-1 Prevents Plasma Cell Differentiation and Prolongs Survival in RK mice. **a** Percentages of CD138^+^B220^low^ cells of splenocytes isolated from aged RK-BL^KO^ mice (*n* = 5) and control mice (*n* = 3–10). **b** Plasma levels of total anti-ssDNA immunoglobulins of six-month-old RK-BL^KO^ mice (*n* = 8) and aged-matched control mice (*n* = 3–6) from two independent experiments using the Alpha Diagnostic International Autoimmunity ELISA kits. **c** Plasma levels of total anti-dsDNA immunoglobulins of six-month-old RK-BL^KO^ mice (*n* = 8) and aged-matched control mice (*n* = 3–6) from two independent experiments using the Alpha Diagnostic International Autoimmunity ELISA kits. **d** Kaplan-Meier overall survival plot of RK-BL^KO^ (*n* = 9) and control mice (WT: *n* = 8, RK: *n* = 11, BL^KO^: *n* = 7). **e** Macroscopic appearance of representative spleens (SP), inguinal lymph nodes (iLN) and mesenteric lymph nodes (mLN) from RK-BL^KO^ and BL^KO^ mice at final endpoint. **f** Macroscopic appearance of representative kidneys from RK-BL^KO^ and RK mice at final endpoint. **g** Percentages of CD19^+^ B cells (including CD19^+^CD5^+^ cells) of living splenocytes isolated from RK-BL^KO^ mice (*n* = 8) and control mice (*n* = 7–11) when euthanasia was required. **h** Percentages of CD19^+^CD5^+^ cells of living splenocytes isolated from RK-BL^KO^ mice (*n* = 8) and control mice (*n* = 7–11) when euthanasia was required. Statistical analysis was performed using one-way ANOVA with Tukey correction for multiple comparison. *P* values are indicated in respective graphs. All data are presented as mean ± standard deviation.

Given that RANK^K240^expression has been shown to promote plasma cell differentiation both in vivo and in vitro^27,34^, we confirmed that Blimp-1 deletion effectively impedes plasma cell differentiation in RK mice. As expected, CD138^+^B220^low^ plasma cells were nearly undetectable in RK-BL^KO^ mice (Fig. 5a, Suppl. Figure 5a), leading to a complete absence of autoantibodies (Fig. 5b, c). Additionally, we observed a significant reduction in IgG2a, IgG2b, and IgM levels in the plasma of both RK-BL^KO^ and BL^KO^ mice compared to RK mice (Suppl. Figure 5b-g). These findings confirm that enforced RANK expression does not compensate for the loss of Blimp-1 and demonstrate that RK-BL^KO^ mice are a valuable model for studying CLL progression in the absence of autoreactive plasma cells and with significantly reduced autoimmune inflammation.

To examine the effects of Blimp-1 loss in RK mice, we monitored a cohort of RK-BL^KO^ mice and their respective controls over time. RK-BL^KO^ mice exhibited prolonged survival compared to RK mice (*p* value = 0.0005, Log-Rank test), with only a subset of RK-BL^KO^ and BL^KO^ animals showing signs of splenomegaly or lymphadenopathy (Fig. 5d, e; Suppl. 5h, i). As expected, autoimmune-mediated kidney damage was prevented by Blimp-1 depletion in RK-BL^KO^ mice (Fig. 5f). Given that depletion of Blimp-1 alone can induce B cell lymphoproliferative disorders^35^, we analyzed CLL levels and total B cell counts in animals reaching endpoint criteria. We did not observe any signs of B cell lymphoproliferation upon Blimp-1 deletion, as total B cell percentages were reduced in the spleens and mesenteric lymph nodes of RK, BL^KO^, and RK-BL^KO^ mice compared to aged WT controls (Fig. 5g, Suppl. Figure 5j). Immune profiling revealed subtle changes in the absence of Blimp-1 compared to RK mice, including a lower percentage of CD8^+^ T cells with a shift from a naïve to an effector phenotype and an increase in Ly6G^+^Ly6C^+^ MDSCs in RK-BL^KO^ compared to BL^KO^ cells (Suppl. Figure 6a-k). While these findings are potentially noteworthy, the observed differences do not fully explain the reduced expansion observed upon Blimp-1 deletion in our model. While we observed increased CLL levels in the spleen and mesenteric lymph nodes of both RK and RK-BL^KO^ mice, the overall percentage of CLL cells at the time of death – occurring significantly later in RK-BL^KO^ mice - did not differ between the two groups (Fig. 5h, Suppl. Figure 5k). Given the initial expansion in the B1/CLL compartment in the absence of Blimp-1, RK-mediated CLL progression appears to be almost completely halted without autoantibody-mediated inflammation. Importantly, these findings suggest that the loss of Blimp-1 mitigates long-term CLL progression in this model, likely due to the absence of a proinflammatory, autoimmune-driven microenvironment.

## Discussion

In this study, we conducted a comprehensive analysis of the RK mouse model, which mirrors both autoimmune and CLL phenotypes, and identified a critical role for plasma cell-mediated autoimmunity in driving CLL progression. Given the clear association of autoimmunity with poor disease prognosis, these findings are clinically relevant. Gaining further insight into the key driving factors of this process could help guide therapeutic strategies for this subset of patients.

By comparing the transcriptomes of CLL cells derived from RK (RK^CLL^) and TCL1-driven models (TC^CLL^), we observed that RK^CLL^ exhibits a pro-inflammatory response signature, whereas TC^CLL^ shows enhanced proliferative signatures. These distinct disease phenotypes can be attributed to differences in the oncogenic strength of the driving factors. In TCL1-driven CLL, the potent oncogene TCL1, coupled with an autoreactive BCR recognizing phosphatidylcholine or other autoantigens, is sufficient to initiate and drive aggressive disease progression, independent of additional autoimmune-derived environmental stimuli. In contrast, the less potent RANK^K240E^ oncogene appears to rely on the pro-inflammatory autoimmune environment to promote the full expansion of autoreactive B cell clones and drive disease progression. This dependency on autoimmune stimulation is reflected in the indolent and chronic phenotype characteristic of the RK model. These findings highlight the complementary roles of oncogenic potential and autoimmune-derived factors in shaping the trajectory of CLL and provide insights into the differential mechanisms underlying disease development in these two models.

To gain insight into the selective expansion of potentially autoreactive subclones within the B1/CLL and plasma cell compartments in RANK^K240E^expressing B cells, we analyzed BCR sequences in sorted B cell populations. We identified stereotyped BCRs, a hallmark of patient-derived CLL cells^10,36,37^, and detected several recurrent CDR3 sequences from the immunoglobulin heavy chain gene in RK mice that were previously described in B1a cells from wild-type mice and CLL cells in different murine disease models^38–40^. Notably, the most frequently observed light chain CDR3 sequence, CALWYSNHWVF, has been implicated in dual-reactive B cells expressing two light chains in the autoreactivity-prone MRL mouse model^41^. This light chain is known for recognizing the Smith antigen, a component of the small nuclear ribonucleoprotein (snRNP) complex, which is commonly targeted in systemic autoimmune diseases like lupus^42^. The recurrence of this sequence across B cell subsets in multiple RK mice suggests antigen-driven clonal expansion, potentially mediated by the Smith antigen. Importantly, these expanded clones were shared between CLL and plasma cell populations within individual mice, and observed across different animals. This supports the hypothesis that a shared (auto)antigen drives both autoimmunity and lymphomagenesis in RK mice. Thus, the expression of active RANK in B cells may hinder the effective elimination or silencing of autoreactive B cell clones, promoting their survival and facilitating the co-development of autoantigen-driven CLL and autoimmunity. Consistent with this, Singh et al. demonstrated that B cells producing pathogenic autoantibodies frequently harbor lymphoma-associated mutations, such as those affecting NF-κB signaling, further linking autoimmunity to lymphoma development^3^.

To investigate the potential impact of autoimmunity on CLL progression, we used a genetic approach to block plasma cell differentiation and thus prevent autoantibody secretion. As B1 cells express low levels of Blimp-1, we evaluate potential cell-intrinsic effects. Our analysis of younger RK-BL^KO^ mice revealed expanded CLL populations, indicating that Blimp-1 deletion does not impair CLL initiation or early expansion. Moreover, viability assays comparing CLL cells from RK and RK-BL^KO^mice showed no differences in cell survival, suggesting that Blimp-1 deficiency does not lead to increased cell turnover or apoptosis in CLL cells. These findings are consistent with studies showing that Blimp-1 deletion in B1 cells impacts antibody production but not their development, expansion, or self-renewal capacity^43^. Additionally, Blimp-1 deletion has been described to drive B cell transformation and for CLL, an association between low Blimp-1 expression and increased CLL aggression is described, further supporting the notion that Blimp-1 deletion does not suppress the intrinsic expansion potential of CLL cells. While these results and literature provide strong evidence against a CLL-intrinsic effect of Blimp-1 deletion, we acknowledge that very subtle or long-term consequences cannot be entirely excluded.

However, we show that our strategy of Blimp-1 deletion was highly effective in inhibiting antibody formation, including autoantibodies. Interestingly, in aged mice, this prevention of autoimmunity reduced CLL progression and prolonged survival. However, when analyzed at six-eight months of age, RK-mediated B1/CLL expansion was higher in mice with Blimp-1 knockout. This observation can be explained by several factors. First, as indicated above, Blimp-1 is a known tumor suppressor in B cells, particularly in diffuse large B-cell lymphoma^35^. While the combination of Blimp-1 deletion and the DLBCL-associated RANK mutation could have potentially induced an aggressive DLBCL phenotype, this was not observed. Instead, we saw a moderate but consistent increase in the B1/CLL cell compartment, suggesting that additional genetic or environmental “hits” are required for full transformation. One possibility for the moderate accumulation of B1 cells is that Blimp-1 plays a role in the negative selection of autoreactive B cells, and its deletion promotes the B1 cell lineage and autoreactive B cell formation, especially when combined with deletion of Pten, a negative regulator of PI3K signaling. Given that the RANK^K240E^mutation also induces elevated PI3K signaling^27^, a similar phenotype may be at play in our model. Thus, our findings confirm that Blimp-1 induction is a crucial mechanism for controlling B1 cell expansion, particularly in the context of constitutive BCR and/or coreceptor signaling. However, Blimp-1 deletion alone is insufficient to drive full transformation or long-term CLL progression in the RK model. Identifying the factors necessary to fully exploit Blimp-1’s tumor-suppressive role will be critical for understanding its function in B cell lymphomagenesis. This is relevant for DLBCL as decreased expression of Blimp-1 is common and a known driver event and has been associated with poor prognosis^44–46^.

Regardless of the initial boost in B1/CLL cell development, in the absence of Blimp-1 (and therefore autoantibody mediated inflammation), CLL progression was almost completely halted in RK-BL^KO^mice, with stable B1/CLL cell levels ranging around 20–30% in the spleen at six-eight months and when mice became moribund at a median age of two years. We analyzed the immune cell compartment as CLL cells have been reported to secrete immunoglobulin M (IgM) to shape the microenvironment in favor of immunosuppression in CLL mouse models by inducing MDSCs, reducing T cell populations, and consequently decreasing survival^47^. In line with previous reports, we detected very low levels of secreted IgM in the serum in the absence of Blimp-1^31,48^. Interestingly, although IgM secretion was absent, we observed that Blimp-1 deletion promoted the formation of an immunosuppressive microenvironment, characterized by the expansion of MDSCs and altered T cell dynamics. This suggests that while Blimp-1 deletion suppresses IgM secretion, MDSC formation still occurred in RK-BL^KO^ mice, suggesting that other mechanisms may contribute to the long-term stability of the CLL population. The lack of soluble inflammatory factors, typically driven by autoantibodies, may explain the long-term stability of the CLL population in RK-BL^KO^mice, as the absence of these pro-inflammatory signals could limit further disease progression. In our study, we observed upregulation of TNF-α-NF-κB signatures in RK-derived CLL, along with modestly elevated levels of TNF-α in the serum of RK mice, suggesting a significant role for this cytokine in promoting CLL progression within the context of autoimmune inflammation. While serum TNF-α levels are widely recognized as biomarkers for inflammation and strongly correlate with the severity of inflammatory responses and the presence of autoimmunity^49–51^, it is important to consider that local TNF-α concentrations in the tumor microenvironment may be significantly higher than what is reflected by serum levels. This localized elevation could contribute more directly to CLL progression by enhancing the pro-inflammatory and immunosuppressive milieu within affected tissues. In CLL, TNF-α can bind directly to its receptor on CLL cells, activating the NF-κB pathway, which enhances survival, proliferation, and resistance to apoptosis in malignant B cells^52,53^. Additionally, TNF-α signaling fosters an inflammatory microenvironment conducive to CLL expansion, both by directly stimulating malignant cells and by modulating the surrounding immune landscape^54^. However, further analysis is needed to determine whether the elevated TNF-α levels observed in RK mice contribute significantly to CLL progression. While TNF-α blockade has been effective in reducing inflammation and improving clinical outcomes in autoimmune diseases, it is important to note that some patients treated with selective TNF inhibitors may develop autoantibodies^55,56^. Therefore, further research is necessary to identify which CLL patients would benefit most from this approach, as combination therapies that include TNF-α inhibitors and Rituximab have shown promising results^57^.

In conclusion, our study demonstrates that autoantibody-mediated inflammation can promote CLL progression in our indolent disease model. We provide new insights into the clonal relationship between B cell populations, showing that both CLL cells and plasma cells contributing to autoimmunity arise from common expanding autoantigen-specific clones. By depleting Blimp-1 and preventing plasma cell formation, we observed an initial expansion of the B1/CLL population, likely due to impaired negative B cell selection processes. However, these factors alone were insufficient to drive long-term disease progression in the absence of autoantibody-promoted inflammation. Our findings suggest that sustained CLL progression is primarily driven by autoinflammatory responses, highlighting the critical role of the inflammatory microenvironment in the long-term expansion of CLL.

## Methods

### Mice

 To genetically remove the Prdm1 gene, we crossed RANK^K240E^mice (RK)^27^ with the Prdm1^fl/fl^-transgenic mouse model^31^ to produce triple-transgenic offspring designated as RANK^K240E^ Prdm1^fl/fl^ CD19^Cre^ (RK-BL^KO^). Control groups included Prdm1^fl/fl^ CD19^Cre^ (BL^KO^) and CD19^Cre^(WT), which also included wild-type littermates. To compare the transcriptome of leukemic cells, RK and Eµ-TCL1 mice (TC)^28^were utilized. For further transcriptional comparison and Blimp-1 protein levels, plasma cells from TC-RK mice^34^ served as a positive control. BL^KO^ were obtained from Jackson Laboratory and TC mice were kindly provided by C. Croce (Ohio State University, Columbus, OH). Mice were euthanized using a two-step process involving isoflurane anaesthesia followed by cervical dislocation. Animals were housed under specific-pathogen-free (SPF) conditions, unlike a previous RK colony that was kept under less stringent hygiene standards. All animal experiments were performed in accordance with ARRIVE guidelines and adhered to German Federal Animal Protection Laws and were approved by the Institutional Animal Care and Use Committee at the Technical University of Munich.

### Flow Cytometry

 Flow cytometry was utilized to analyze peripheral blood and organs obtained from mice. Single-cell suspensions were prepared by passing organs through a 100 μm cell strainer in PBS, and erythrocytes were lysed using G-DEXTMIIb RBC Lysis Buffer from Intron Biotechnologies. To exclude dead cells, either Zombie Aqua™ Fixable Viability Kit from BioLegend^®^ or DAPI staining at a concentration of 1 µg/ml (Sigma Aldrich) was employed. To block free Fc receptors, either anti-mouse CD16/32 from BioLegend^®^ was used. Cells were then stained with fluorochrome-labeled antibodies following the manufacturer’s instructions and listed in the Supplementary Methods. For intracellular Blimp-1 detection, fixation and permeabilization was performed using the eBioscience™ Intracellular Fixation & Permeabilization Kit according to the manufacturer’s protocol.

### Histology

 After fixation in 10% neutral buffered formalin for 48 h, murine kidneys were paraffin-embedded, sectioned and stained with Hematoxylin-Eosin (HE) following standard procedures. Slides were scanned using a Leica AT2 biosystem and a board-certified pathologist conducted analysis and evaluation.

### Measurement of immunoglobulins and autoantibodies

 We utilized the LEGENDplex™ Mouse Immunoglobulin Isotyping Panel and Mouse Inflammation Panel manufactured by BioLegend^®^ to detect plasma immunoglobulins and TNF-α levels. The assay was conducted following the manufacturer’s instructions. Quantification of total murine anti-ssDNA and anti-dsDNA immunoglobulins in plasma was performed using the corresponding Elisa Kits from Alpha Diagnostic International according to the manufacturer’s protocol.

#### RNA Sequencing

 For differential expression and BCR comparison, CLL cells from TC and RK mice were isolated using magnetic-activated cell sorting with the murine B1a cell isolation kit, B cells from WT mice were isolated using murine CD19 MicroBeads, CD138^+^plasma cells from RK mice and TC-RK mice were additionally isolated using CD138 MicroBeads from Miltenyi Biotec, all from Miltenyi Biotec, according to the manufacturer’s protocol. RNA isolation, sequencing and analysis were performed as previously described^34^. In brief, the reference genome GRCm38.p6 was used for alignment. Differential expression analysis was performed in R (v4.2.1) with DESeq2 (v1.36)^58^. A gene was deemed differentially expressed if its adjusted p-value was below 0.05. Gene set enrichment analysis was conducted in R (v4.2.1) using the GSEA package (v4.3.2). A pathway was considered to be significantly deregulated if the FDR value was below 0.05. Genes contributing to different hallmarks in the GSEA, and Blimp-1 target genes, are presented in separate heatmaps, displaying z-transformed expression data. For further analysis please refer to the Supplementary Methods.

#### Statistics

 Statistical significance was assessed using paired or unpaired two-tailed Student’s *t*-tests, one-way ANOVA, or Log-Rank (Mantel-Cox) tests, as appropriate, with Prism Version 8.0 from GraphPad Software Inc. Results are shown as the mean ± SD unless otherwise specified, and *p* values are depicted within each graph.

## Electronic supplementary material

Below is the link to the electronic supplementary material.

Supplementary Material 1

Supplementary Material 2

Supplementary Material 3

## Data Availability

All data required to evaluate the conclusions of this paper are included within the manuscript and/or provided as Supplementary Information. Raw data will be uploaded to the ENA database upon manuscript acceptance and are available to reviewers upon request from the corresponding author.
