# Supplementary material for: Autoimmunity promotes chronic lymphocytic leukemia progression in an indolent disease model
Source: Sci Rep. 2025 Feb 3;15:4117. doi: 10.1038/s41598-025-86876-1 (PMC11791097; doi:10.1038/s41598-025-86876-1)
Supplement: Supplementary file 3 — Supplementary Material 3 [file 41598_2025_86876_MOESM3_ESM.pdf]

Autoimmunity promotes CLL progression in an indolent disease model

Lisa Pfeuffer[1,2], Viola Siegert[1,2], Riccardo Trozzo[2,3], Katja Steiger[4,5], Roland Rad[2,3,5], Jürgen Ruland[1,2,5,6], Maike Buchner[1,2]

[1] *Institute of Clinical Chemistry and Pathobiochemistry, TUM School of Medicine and Health, Technical University of Munich, Munich, Germany*
[2] *Center for Translational Cancer Research (TranslaTUM) and TUM University Hospital, Technical University of Munich, Munich, Germany*
[3] *Institute of Molecular Oncology and Functional Genomics, TUM School of Medicine and Health, Technical University of Munich, 81675 Munich, Germany*
[4] *Institute of Pathology Technical University Munich, Munich, Germany,*
[5] *German Cancer Consortium (DKTK), partnership between German Cancer Research Center (DKFZ) and TUM University Hospital, Munich, Germany.*
[6] *German Center for Infection Research (DZIF), Munich partner site, Munich, Germany.*

SUPPLEMENTARY DATA

SUPPLEMENTARY METHODS

Antibodies for flow cytometric analysis. The following antibodies were used for surface staining of cell suspensions for flow cytometric analysis according to manufacturer's protocols from BioLegend®: anti-CD19-APC-Cy7 (clone 5D3, #11553), anti-CD5-PE (clone 53-7.3, #100607), anti-CD3-PE (clone 145-2C11, # 100308), anti-CD138-PE (clone 281-2, #142504), anti-CD45R-APC-Cy7 (RA3-6B2, #103224), anti-CD23-PE-Cy7 (B3B4, #101614), anti-CD25-PE (PC61.5, #102008), anti-CD19-APC (6D5, #115512), anti-Ly6C-APC (clone HK1.4, #128016), anti-Ly6G-PerCP-Cy5.5 (clone 1A8, # 127615), anti-CD11b-BV421 (clone M1/70, # 101235), anti-CD4-APC (clone GK1.5, #100412), anti-CD44-PE-Cy7 (clone IM7, #103029), anti-CD8-PerCP-Cy5.5 (clone 53-6.7, #100734), anti-TCRβ-BV421 (clone H57-597; # 109230) and anti-Blimp-1-AF 647 (clone 5E7, #150004). Anti-CD21-PE (7G6, #552957) was purchased from BD. Following antibodies were purchased from eBioscience and used according to the manufacturer's protocol: anti-IgM-PE-Cy7 (clone II/41, #25-5790-81), anti-CD62L-PE (clone MEL-14, #12-0621-81), anti-CD45R-eFluor450 (RA3-6B2, #48-0452-82), anti-CD5-APC (53-7.3, #17-0051-82), anti-CD117-APC (2B8, #17-1171-81).

RNA Sequencing analysis. For BCR repertoire analysis, the TRUST4 open-source algorithm was applied to reconstruct IGH and IGL chains for each sample[1]. Clonality plots were generated

based on the method described by Weber et al. 2019[2], with some modifications. In brief, the network was constructed using the Fruchterman-Reingold layout, where each dot represents a clone. The size of each dot corresponds to a scaled value of the cube root of the read count. A link between two clones indicates identical V and J regions with a 1bp difference in the CDR3 sequence. Clusters of clones representing 10% or more of the total read counts for a sample were highlighted with color.

SUPPLEMENTARY FIGURE LEGENDS

SUPPLEMENTARY FIGURE 1: Differential Gene Expression in Splenic CLL cells from RK and TC mice

a Heatmap analysis of differentially regulated genes associated with the most significantly altered hallmarks identified by gene set enrichment analysis (GSEA) analysis of splenic CLL cells isolated from aged RK mice (RK^{CLL}, orange; n = 4) and TC mice (TC^{CLL}, light orange; n = 3). The color scale represents the Z score, with red indicating upregulation and blue indicating downregulation.

SUPPLEMENTARY FIGURE 2: Characteristics of TC and RK mice

a Plasma levels of total anti-ssDNA immunoglobulins of diseased TC mice (n = 4) and aged control mice (n = 12) from two independent experiments using the Alpha Diagnostic International Autoimmunity ELISA kits**. b** Plasma levels of total anti-dsDNA immunoglobulins of diseased TC mice (n = 4) and aged control mice (n = 14) from two independent experiments using the Alpha Diagnostic International Autoimmunity ELISA kits. **c** *Igl* and *Igk* clonality analysis from of $CD19^{+}CD5^{+}$ CLL cells and $CD138^{+}$ plasma cells (Pc) isolated from four different five- to six-month-old RK mice. Statistical analysis was performed using unpaired Student's *t* test for comparison of two groups. *P* values are indicated in respective graphs. All data are presented as mean ± standard deviation.

SUPPLEMENTARY FIGURE 3: Loss of Blimp-1 has Minor Effects on Early B Cell Development in the Bone Marrow of RK mice

a Representative flow cytometric analysis of intracellular Blimp-1 expression in RK-derived B220^{+}CD5^{+} CLL cells (RK^{CLL}, orange) compared to B220^{+}CD5^{neg} B (RK^{Bc}, light blue) and plasma cells (RK^{Pc}, black) from the same model (top), with geometric mean values shown in the dot plot below (n = 5). The CD19^{+} B cell pool (RK-BL^{Bc+CLL}, grey), including CLL cells, from the RK-BL^{KO} model served as a negative control, while plasma cells (TC-RK^{Pc}, green) from the TC-RK mouse model[3] served as a positive control. **b** Heatmap analysis of Blimp-1 target gene expression in CLL cells (RK^{CLL}) isolated from aged RK mice (n = 4) and CD19^{+} WT B cells (WT^{Bc}, n = 4) compared to plasma cells (TC-RK^{Pc}) from TC-RK mice (n = 4). The color scale represents the Z score, with red indicating upregulation and blue indicating downregulation. **c** Percentages of B220^{+}CD19^{+} cells of living cells isolated from bone marrow of three- to six-month-old RK-BL^{KO} (n = 10) and aged-matched controls (n = 5 – 7). **d** Representative flow cytometric analysis of B220 and IgM surface expression was performed on pre-gated B220^{+}CD19^{+} bone marrow cells from three- to six-month-old RK-BL^{KO} mice and age-matched controls. Immature B cells were characterized by low B220 and positive IgM expression, while recirculating B cells exhibited high B220 and IgM expression. IgM-negative cells (IgM^{neg}), identified by low B220 and absent IgM expression, were further assessed for CD25 and c-Kit expression. Pro B cells were defined as c-Kit^{+}CD25^{neg}, and pre B cells as c-Kit^{neg}CD25^{+}. **e** Percentages of pro B cells within the IgM^{neg} cell compartment in bone marrow of three- to six-month-old RK-BL^{KO} (n = 10) and aged-matched controls (n = 5 – 7). **f** Percentages of pre B cells within the IgM^{neg} cell compartment in bone marrow of three- to six-month-old RK-BL^{KO} (n = 10) and aged-matched controls (n = 5 – 7). Statistical analysis was performed using one-way ANOVA with Tukey correction for multiple comparison. *P* values are indicated in respective graphs. All data are presented as mean ± standard deviation.

SUPPLEMENTARY FIGURE 4: Impact of Blimp-1 Deletion on CLL Cell proportion and CLL Cell Survival in RK-BL^{KO} mice

a Representative flow cytometric analysis of CD19 and CD5 surface expression on splenocytes pre-gated on live cells isolated from six-month-old RK-BL^{KO} and BL^{KO} control mice. Gating of CD19^{+}CD5^{+} CLL cells and CD19^{+}CD5^{neg} B cells is shown in the left panel. CD19^{+}CD5^{neg} B cells were further characterized by CD21 and CD23 expression into marginal zone B cells (MZ, defined as CD21^{+}CD23^{neg}) and follicular B cells (FO, defined as CD21^{low}CD23^{+}) in the right panel. **b** The effect of Blimp-1 deletion on the survival of MACS-isolated CD19^{+} B cells including CLL cells, from RK-BL^{KO} (n = 4) compared to RK mice (n =4), measured in technical duplicates by flow cytometry over time relative to the day of isolation. Data are presented as

mean of biological replicates ± standard deviation and were pooled from two independent experiments. **c** Relative percentage of CLL cells in MACS-isolated total $CD19^{+}$ B cells from RK-BL^{KO} (n = 4) compared to RK mice (n = 4), measured in technical duplicates by flow cytometry over time relative to the day of isolation. Data are presented as mean of biological replicates ± standard deviation and were pooled from two independent experiments.

SUPPLEMENTARY FIGURE 5: $RANK^{K240E}$ Cannot Compensate for Loss of Blimp-1 During Plasma Cell Differentiation

a Representative flow cytometric analysis of CD138 and B220 surface expression on splenocytes pre-gated on live cells isolated from aged RK-BL^{KO} and control mice. Gating of $CD138^{+}B220^{low}$ plasma cells is shown. **b** Quantification of IgG1 levels in plasma samples from six-month-old RK-BL^{KO} (n = 8) and control mice (n = 3 -6) determined by flow cytometry-based multiplex immunoassay. Pooled data from two independent experiments. **c** Quantification of IgG2a levels in plasma samples from six-month-old RK-BL^{KO} (n = 8) and control mice (n = 3 -6) determined by flow cytometry-based multiplex immunoassay. Pooled data from two independent experiments. **d** Quantification of IgG2b levels in plasma samples from six-month-old RK-BL^{KO} (n = 8) and control mice determined by flow cytometry-based multiplex immunoassay. Pooled data from two independent experiments. **e** Quantification of IgG3 levels in plasma samples from six-month-old RK-BL^{KO} (n = 8) and control mice (n = 3 - 6) determined by flow cytometry-based multiplex immunoassay. Pooled data from two independent experiments. **f** Quantification of IgM levels in plasma samples from six-month-old RK-BL^{KO} (n = 8) and control mice (n = 3 -6) determined by flow cytometry-based multiplex immunoassay. Pooled data from two independent experiments. **g** Quantification of IgA levels in plasma samples from six-month-old RK-BL^{KO} (n = 8) and control mice (n = 3 -6) determined by flow cytometry-based multiplex immunoassay. Pooled data from two independent experiments. **h** Dot plot graph depicts spleen (SP) weight in gram (g) of RK-BL^{KO} (n = 9) and control mice (n = 7 – 9) when euthanasia was required. **i** Dot plot graph depicts mesenteric lymph node (mLN) weight in gram (g) of RK-BL^{KO} (n = 9) and control mice (n = 7 – 9) when euthanasia was required. **j** Percentages of total $CD19^{+}$ B cells (including $CD19^{+}CD5^{+}$ cells) of living splenocytes isolated from RK-BL^{KO} (n = 6) and control mice (n = 4 - 7) when euthanasia was required. **k** Percentages of $CD19^{+}CD5^{+}$ cells of living splenocytes isolated from RK-BL^{KO} mice (n = 6) and control mice (n = 4 - 7) when euthanasia was required. Statistical analysis was performed using one-way ANOVA with Tukey correction for multiple comparison. *P* values are indicated in respective graphs. All data are presented as mean ± standard deviation.

SUPPLEMENTARY FIGURE 6: Loss of Blimp-1 Does Not Affect the Immune Cell Microenvironment in RK mice

a Representative flow cytometric analysis of CD11b and CD3 surface expression on splenocytes pre-gated on CD19^{neg}GFP^{neg} cells isolated from six-month-old RK-BL^{KO} and BL^{KO} control mice. **b** Percentages of CD3^{+} cells pre-gated on CD19^{neg}GPF^{neg} cells isolated from splenocytes of six-month-old RK-BL^{KO} (n = 5) and aged-matched controls (n = 3 – 8). **c** Percentages of CD11b^{+} cells pre-gated on CD19^{neg}GPF^{neg} cells isolated from splenocytes of six-month-old RK-BL^{KO} (n = 5) and aged-matched controls (n = 3 – 8). **d** Percentages of CD4^{+} cells pre-gated on TCRβ^{+}GPF^{neg} T cells isolated from splenocytes of six-month-old RK-BL^{KO} (n = 10) and aged-matched controls (n= 3 – 6). **e** Percentages of naïve CD4^{+} cells (defined by CD44^{neg}CD62L^{+} expression) pre-gated on CD4^{+}TCRβ^{+}GPF^{neg} T cells isolated from splenocytes of six-month-old RK-BL^{KO} and aged-matched controls (n= 3 – 6). **f** Percentages of effector CD4^{+} cells (defined by CD44^{+}CD62L^{neg} expression) pre-gated on CD4^{+}TCRβ^{+}GPF^{neg} T cells isolated from splenocytes of six-month-old RK-BL^{KO} (n = 10) and aged-matched controls (n= 3 – 6). **g** Percentages of CD8^{+} cells pre-gated on TCRβ^{+}GPF^{neg} T cells isolated from splenocytes of six-month-old RK-BL^{KO} (n = 10) and aged-matched controls (n= 3 – 6). **h** Percentages of naïve CD8^{+} cells (defined by CD44^{neg}CD62L^{+} expression) pre-gated on CD8^{+}TCRβ^{+}GPF^{neg} T cells isolated from splenocytes of six-month-old RK-BL^{KO} (n = 10) and aged-matched controls (n= 3 – 6). **i** Percentages of effector CD8^{+} cells (defined by CD44^{+}CD62L^{neg} expression) pre-gated on CD8^{+}TCRβ^{+}GPF^{neg} T cells isolated from splenocytes of six-month-old RK-BL^{KO} (n = 10) and aged-matched controls (n= 3 – 6). **j** Percentages of Ly6G^{+}Ly6C^{+} cells pre-gated on CD11b^{+}CD3^{neg} CD19^{neg}GPF^{neg} cells isolated from splenocytes of six-month-old RK-BL^{KO} (n = 5) and aged-matched controls (n = 3 – 8). **k** Percentages of Ly6G^{high} cells pre-gated on CD11b^{+}CD3^{neg} CD19^{neg}GPF^{neg} cells isolated from splenocytes of six-month-old RK-BL^{KO} (n = 5) and aged-matched controls (n = 3 – 8). Statistical analysis was performed using one-way ANOVA with Tukey correction for multiple comparison. *P* values are indicated in respective graphs. All data are presented as mean ± standard deviation.

SUPPLEMENTARY TABLE LEGENDS

Supplementary Table 1 List of commonly deregulated genes in CLL^{RK} (n = 4) and CLL^{TC} (n = 3) cells compared to $CD19^{+}$ WT B cells (n = 4). **Supplementary Table 2** DESeq2 was used to compare MACS-isolated CLL cells from RK (n = 4) and TC (n = 3) mice.

SUPPLEMENTARY REFERENCES

1 Song, L. *et al.* TRUST4: immune repertoire reconstruction from bulk and single-cell RNA-seq data. *Nat Methods* **18**, 627-630 (2021). https://doi.org:10.1038/s41592-021-01142-2

2 Weber, J. *et al.* PiggyBac transposon tools for recessive screening identify B-cell lymphoma drivers in mice. *Nat Commun* **10**, 1415 (2019). https://doi.org:10.1038/s41467-019-09180-3

3 Pfeuffer, L. *et al.* B-cell intrinsic RANK signaling cooperates with TCL1 to induce lineage-dependent B-cell transformation. *Blood Cancer J* **14**, 151 (2024). https://doi.org:10.1038/s41408-024-01123-6

a)

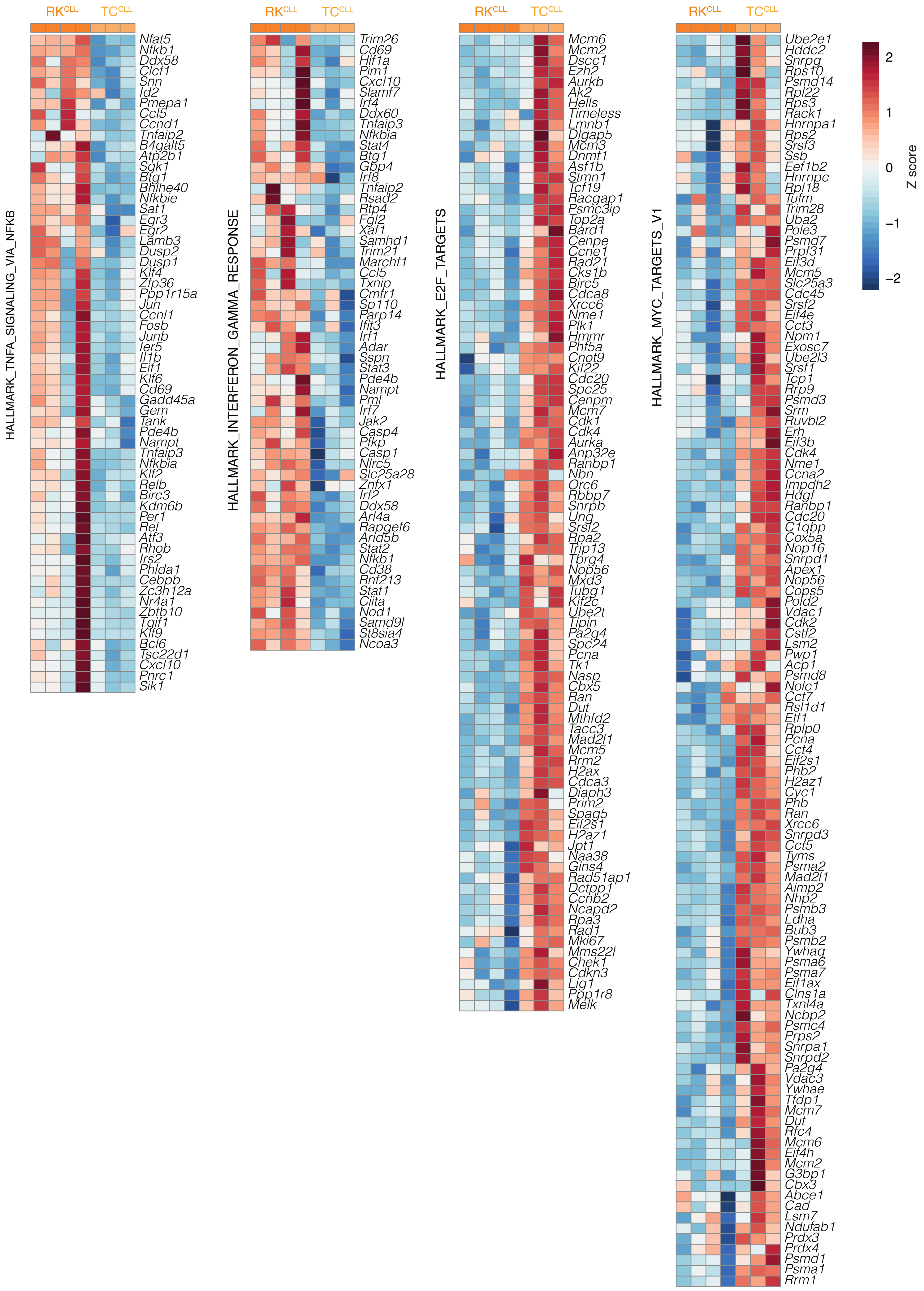
RK^CLL TC^CLL
HALLMARK_TNFA_SIGNALING_VIA_NFKB
Nfat5
Nfkb1
Ddx58
Clcf1
Snn
Id2
Pmepa1
Ccl5
Ccnd1
Tnfaip2
B4galt5
Atp2b1
Sgk1
Btg1
Bhlhe40
Nfkbie
Sat1
Egr3
Egr2
Lamb3
Dusp2
Dusp1
Klf4
Zfp36
Ppp1r15a
Jun
Ccnl1
Fosb
Junb
Ier5
Il1b
Eif1
Klf6
Cd69
Gadd45a
Gem
Tank
Pde4b
Nampt
Tnfaip3
Nfkbia
Klf2
Relb
Birc3
Kdm6b
Per1
Rel
Atf3
Rhob
Irs2
Phlda1
Cebpb
Zc3h12a
Nr4a1
Zbtb10
Tgif1
Klf9
Bcl6
Tsc22d1
Cxcl10
Pnrc1
Sik1
RK^CLL TC^CLL
HALLMARK_INTERFERON_GAMMA_RESPONSE
Trim26
Cd69
Hif1a
Pim1
Cxcl10
Slamf7
Irf4
Ddx60
Tnfaip3
Nfkbia
Stat4
Btg1
Gbp4
Irf8
Tnfaip2
Rsad2
Rtp4
Fgl2
Xaf1
Samhd1
Trim21
Marchf1
Ccl5
Txnip
Cmtr1
Sp110
Parp14
Ifit3
Irf1
Adar
Sspn
Stat3
Pde4b
Nampt
Pml
Irf7
Jak2
Casp4
Pfkp
Casp1
Nlrc5
Slc25a28
Znfx1
Irf2
Ddx58
Arl4a
Rapgef6
Arid5b
Stat2
Nfkb1
Cd38
Rnf213
Stat1
Ciita
Nod1
Samd9l
St8sia4
Ncoa3
RK^CLL TC^CLL
HALLMARK_E2F_TARGETS
Mcm6
Mcm2
Dscc1
Ezh2
Aurkb
Ak2
Hells
Timeless
Lmnb1
Dlgap5
Mcm3
Dnmt1
Asf1b
Stmn1
Tcf19
Racgap1
Psmc3ip
Top2a
Bard1
Cenpe
Ccne1
Rad21
Cks1b
Birc5
Cdca8
Xrcc6
Nme1
Plk1
Hmmr
Phf5a
Cnot9
Cdc20
Spc25
Cenpm
Mcm7
Cdk1
Cdk4
Aurka
Anp32e
Ranbp1
Nbn
Orc6
Rbbp7
Snrpb
Ung
Srsf2
Rpa2
Trip13
Tbrg4
Nop56
Mxd3
Tubg1
Kif2c
Ube2t
Tipin
Pa2g4
Spc24
Pcna
Tk1
Nasp
Cbx5
Ran
Dut
Mthfd2
Tacc3
Mad2l1
Mcm5
Rrm2
H2ax
Cdca3
Diaph3
Prim2
Spag5
Eif2s1
H2az1
Jpt1
Naa38
Gins4
Rad51ap1
Dctpp1
Ccnb2
Ncapd2
Rpa3
Rad1
Mki67
Mms22l
Chek1
Cdkn3
Lig1
Ppp1r8
Melk
RK^CLL TC^CLL
HALLMARK_MYC_TARGETS_V1
Ube2e1
Hddc2
Snrpg
Rps10
Psmd14
Rpl22
Rps3
Rack1
Hnrnpa1
Rps2
Srsf3
Ssb
Eef1b2
Hnrnpc
Rpl18
Tufm
Trim28
Uba2
Pole3
Psmd7
Prpf31
Eif3d
Mcm5
Slc25a3
Cdc45
Srsf2
Eif4e
Cct3
Npm1
Exosc7
Ube2l3
Srsf1
Tcp1
Rrp9
Psmd3
Srm
Ruvbl2
Erh
Eif3b
Cdk4
Nme1
Ccna2
Impdh2
Hdgf
Ranbp1
Cdc20
C1qbp
Cox5a
Nop16
Snrpd1
Apex1
Nop56
Cops5
Pold2
Vdac1
Cdk2
Cstf2
Lsm2
Pwp1
Acp1
Psmd8
Nolc1
Cct7
Rsl1d1
Etf1
Rplp0
Pcna
Cct4
Eif2s1
Phb2
H2az1
Cyc1
Phb
Ran
Xrcc6
Snrpd3
Cct5
Tyms
Psma2
Mad2l1
Aimp2
Nhp2
Psmb3
Ldha
Bub3
Psmb2
Ywhaq
Psma6
Psma7
Eif1ax
Clns1a
Txnl4a
Ncbp2
Psmc4
Prps2
Snrpa1
Snrpd2
Pa2g4
Vdac3
Ywhae
Tfdp1
Mcm7
Dut
Rfc4
Mcm6
Eif4h
Mcm2
G3bp1
Cbx3
Abce1
Cad
Lsm7
Ndufab1
Prdx3
Prdx4
Psmd1
Psma1
Rrm1
2
1
0
−1
−2
Z score

Suppl.Fig. 1

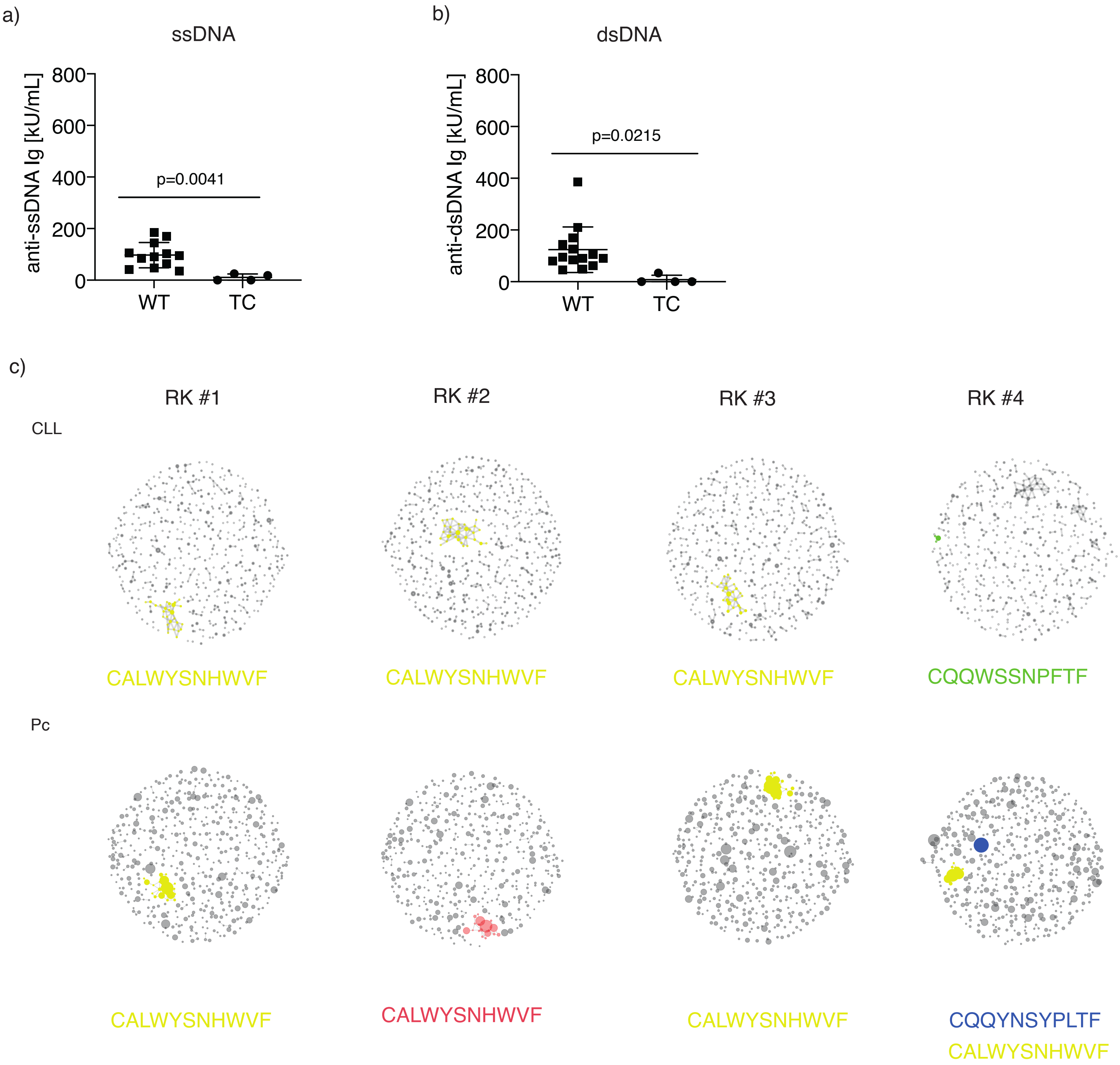
a)
ssDNA
anti-ssDNA Ig [kU/mL]
800
600
400
200
0
p=0.0041
WT
TC
b)
dsDNA
anti-dsDNA Ig [kU/mL]
800
600
400
200
0
p=0.0215
WT
TC
c)
RK #1
RK #2
RK #3
RK #4
CLL
CALWYSNHWVF
CALWYSNHWVF
CALWYSNHWVF
CQQWSSNPFTF
Pc
CALWYSNHWVF
CALWYSNHWVF
CALWYSNHWVF
CQQYNSYPLTF
CALWYSNHWVF

Suppl.Fig. 2

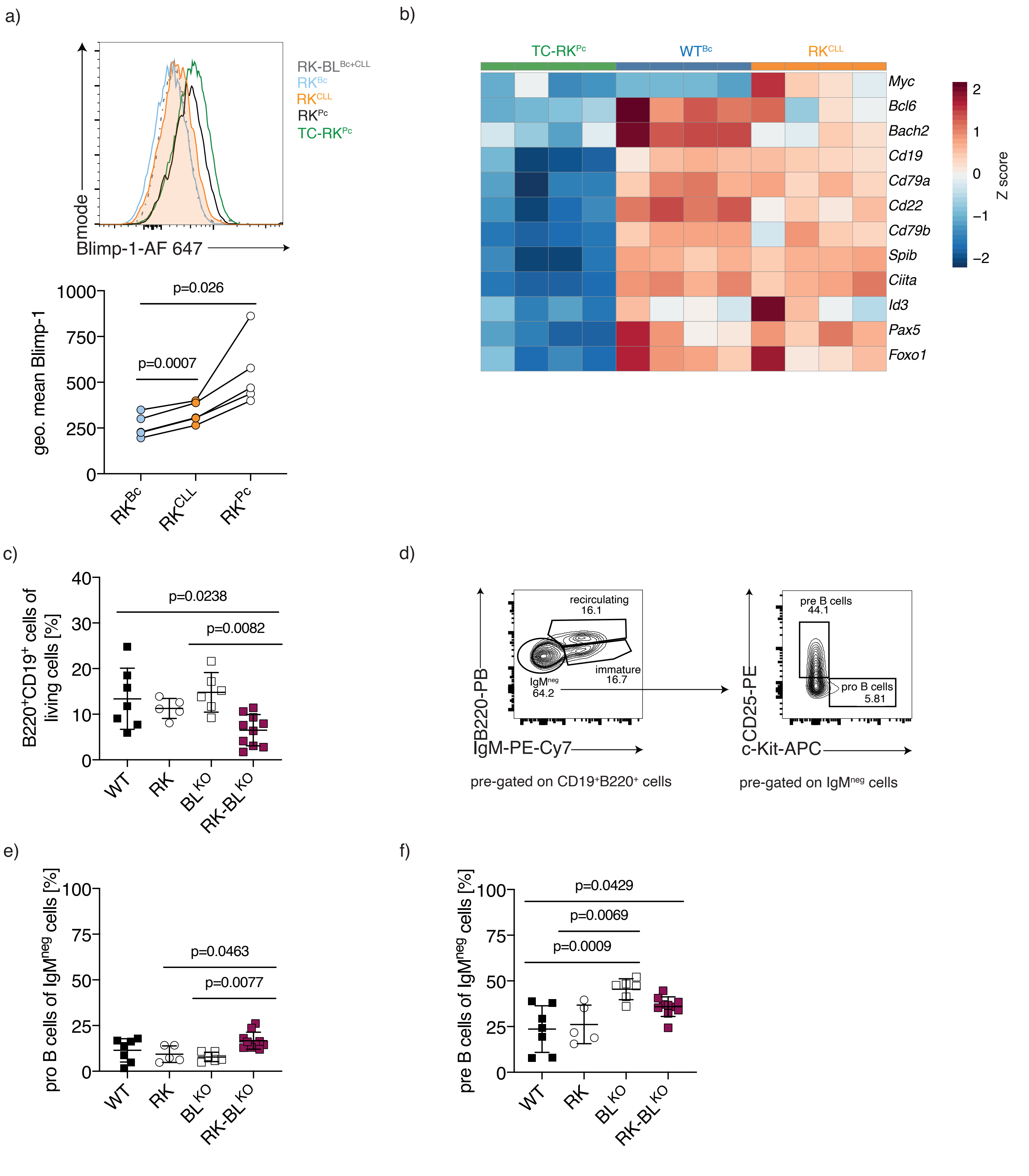
a)
RK-BL Bc+CLL
RK Bc
RK CLL
RK Pc
TC-RK Pc
mode
Blimp-1-AF 647
geo. mean Blimp-1
p=0.026
p=0.0007
1000
750
500
250
0
RK Bc
RK CLL
RK Pc
b)
TC-RK Pc
WT Bc
RK CLL
Myc
Bcl6
Bach2
Cd19
Cd79a
Cd22
Cd79b
Spib
Ciita
Id3
Pax5
Foxo1
2
1
0
−1
−2
Z score
c)
B220+CD19+ cells of living cells [%]
p=0.0238
p=0.0082
40
30
20
10
0
WT
RK
BL KO
RK-BL KO
d)
recirculating 16.1
immature 16.7
IgMneg 64.2
B220-PB
IgM-PE-Cy7
pre-gated on CD19+B220+ cells
pre B cells 44.1
pro B cells 5.81
CD25-PE
c-Kit-APC
pre-gated on IgMneg cells
e)
pro B cells of IgMneg cells [%]
p=0.0463
p=0.0077
100
75
50
25
0
WT
RK
BL KO
RK-BL KO
f)
pre B cells of IgMneg cells [%]
p=0.0429
p=0.0069
p=0.0009
100
75
50
25
0
WT
RK
BL KO
RK-BL KO

Suppl.Fig. 3

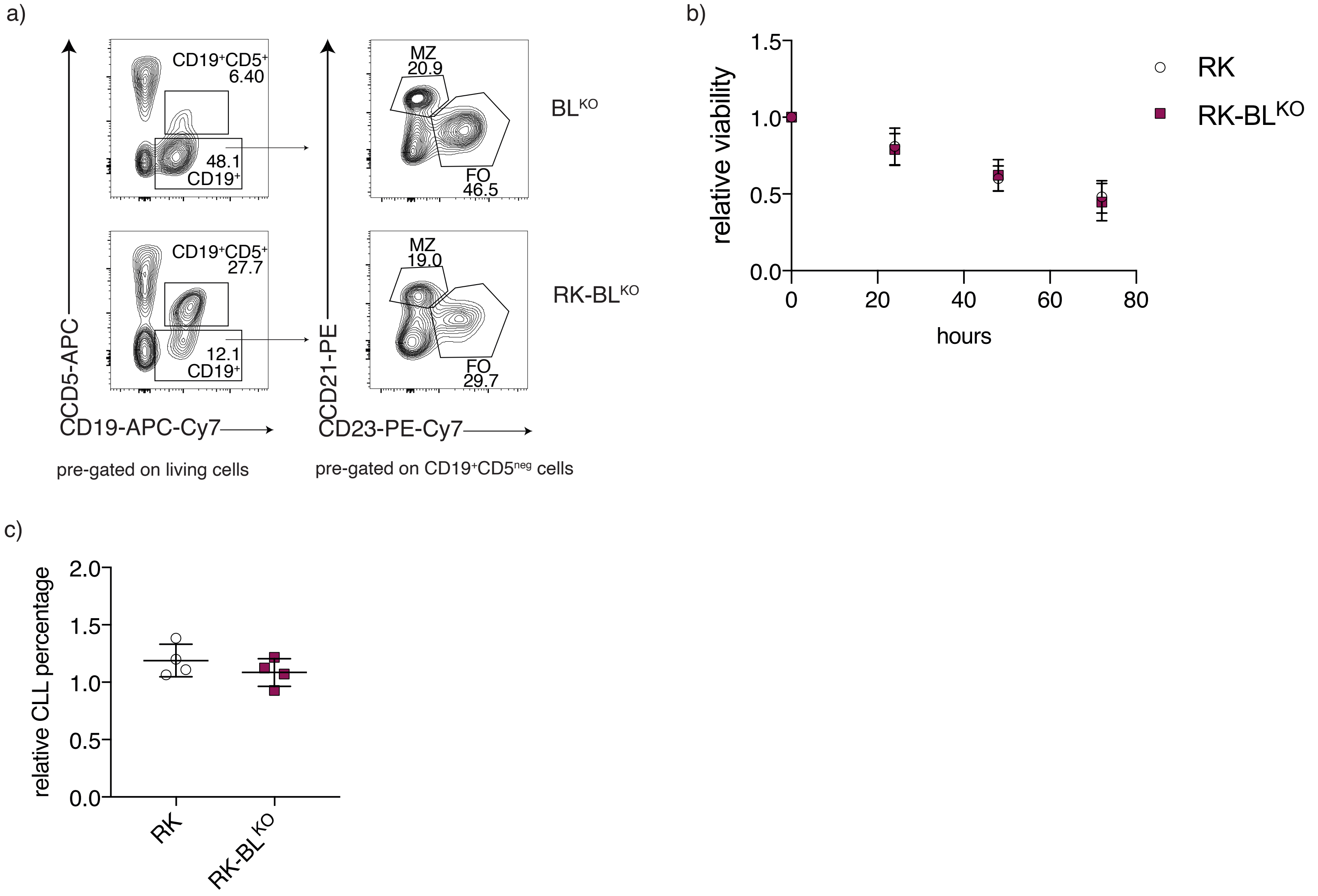
a)
CD19+CD5+
6.40
48.1
CD19+
MZ
20.9
FO
46.5
BLKO
CD19+CD5+
27.7
12.1
CD19+
MZ
19.0
FO
29.7
RK-BLKO
CD5-APC
CD19-APC-Cy7
CD21-PE
CD23-PE-Cy7
pre-gated on living cells
pre-gated on CD19+CD5neg cells
b)
relative viability
hours
RK
RK-BLKO
c)
relative CLL percentage
RK
RK-BLKO

Suppl.Fig. 4

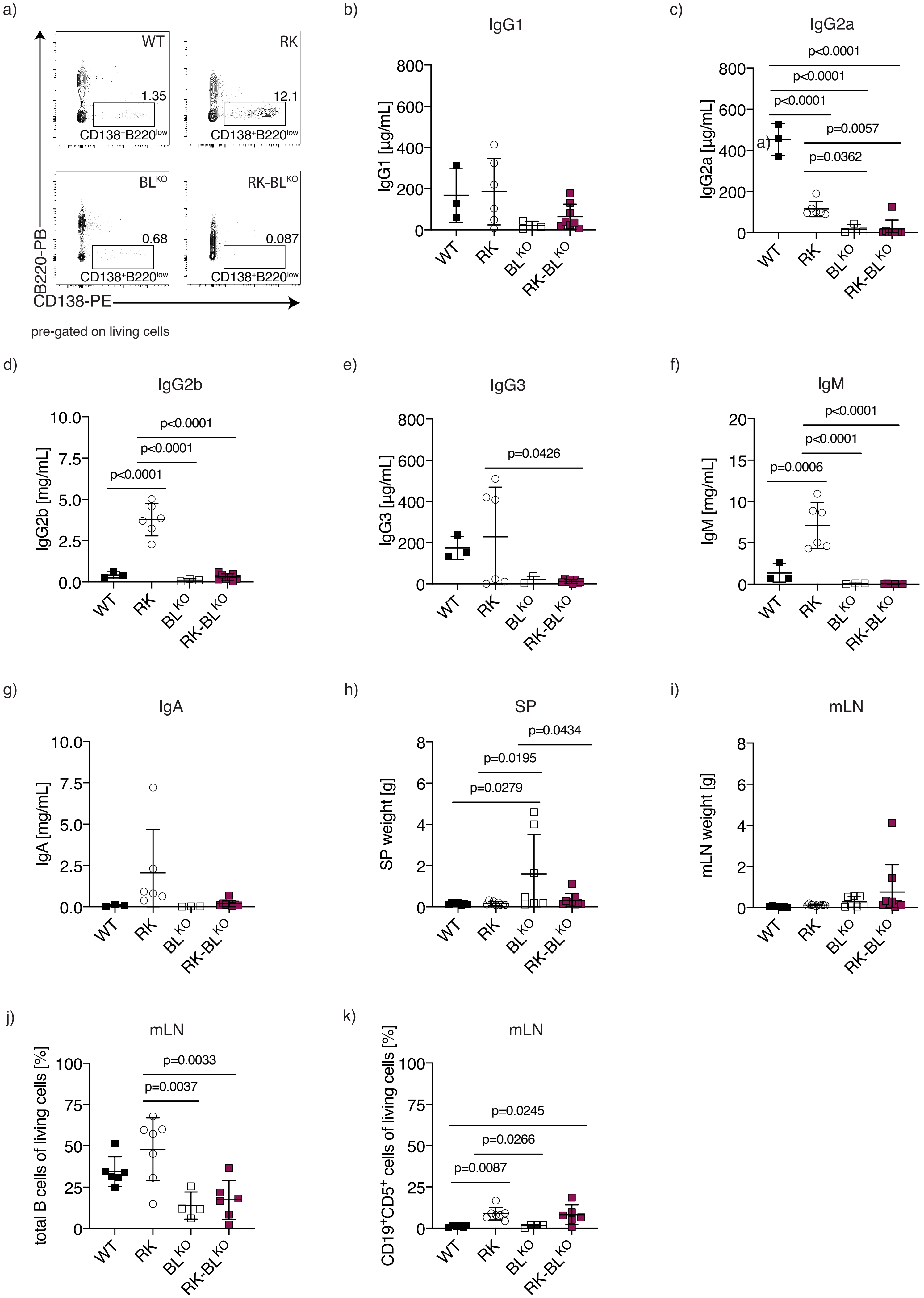
a)
WT
RK
BLKO
RK-BLKO
1.35
12.1
0.68
0.087
CD138+B220low
CB220-PB
CD138-PE
pre-gated on living cells
b)
IgG1
IgG1 [µg/mL]
c)
IgG2a
IgG2a [µg/mL]
p<0.0001
p<0.0001
p<0.0001
p=0.0057
p=0.0362
d)
IgG2b
IgG2b [mg/mL]
p<0.0001
p<0.0001
p<0.0001
e)
IgG3
IgG3 [µg/mL]
p=0.0426
f)
IgM
IgM [mg/mL]
p<0.0001
p<0.0001
p=0.0006
g)
IgA
IgA [mg/mL]
h)
SP
SP weight [g]
p=0.0434
p=0.0195
p=0.0279
i)
mLN
mLN weight [g]
j)
mLN
total B cells of living cells [%]
p=0.0033
p=0.0037
k)
mLN
CD19+CD5+ cells of living cells [%]
p=0.0245
p=0.0266
p=0.0087
WT
RK
BLKO
RK-BLKO

Suppl.Fig. 5

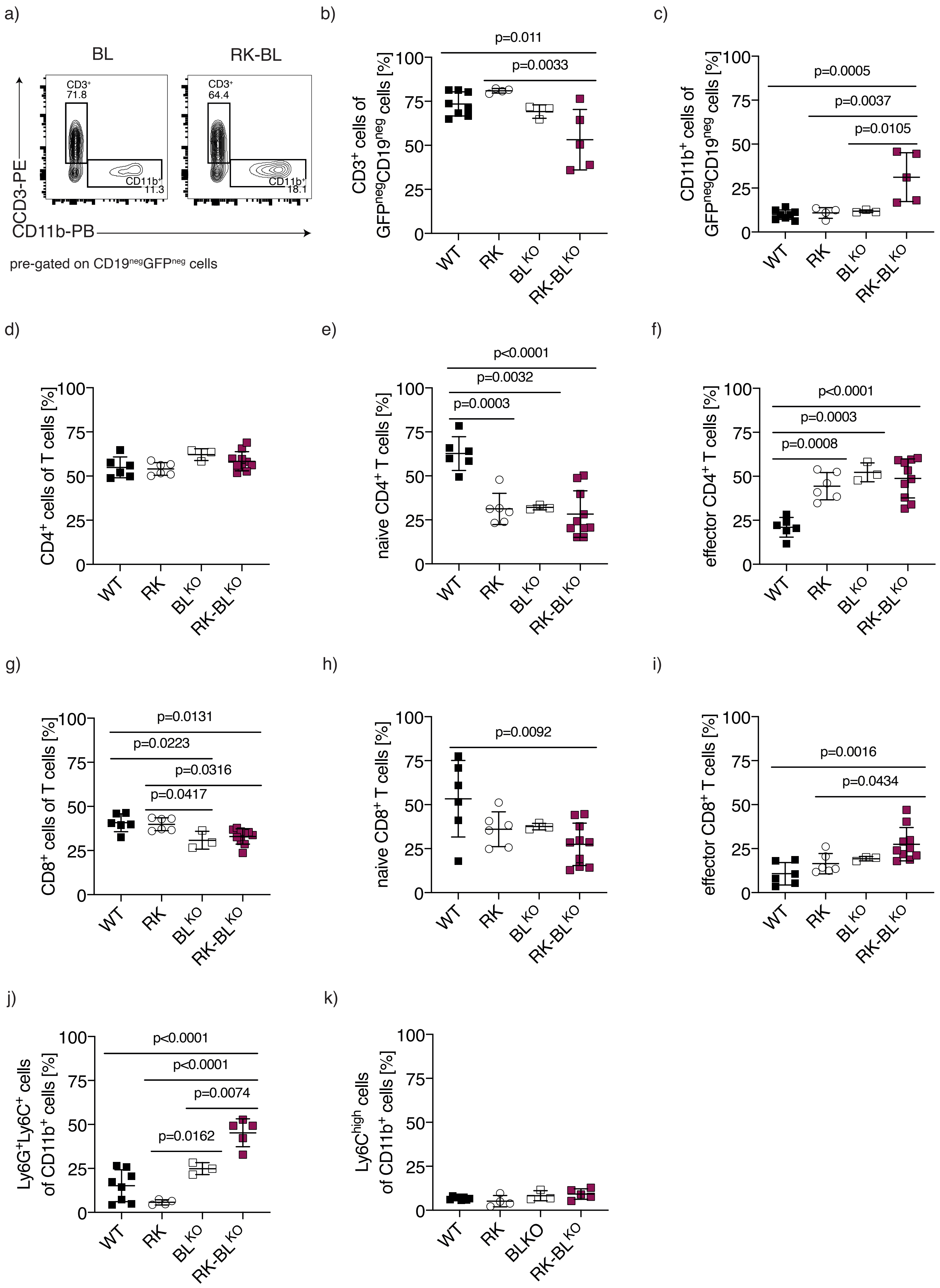

a)
BL
RK-BL
CD3+ 71.8
CD11b+ 11.3
CD3+ 64.4
CD11b+ 18.1
CD3-PE
CD11b-PB
pre-gated on CD19negGFPneg cells
b)
CD3+ cells of GFPnegCD19neg cells [%]
p=0.011
p=0.0033
c)
CD11b+ cells of GFPnegCD19neg cells [%]
p=0.0005
p=0.0037
p=0.0105
d)
CD4+ cells of T cells [%]
e)
naive CD4+ T cells [%]
p<0.0001
p=0.0032
p=0.0003
f)
effector CD4+ T cells [%]
p<0.0001
p=0.0003
p=0.0008
g)
CD8+ cells of T cells [%]
p=0.0131
p=0.0223
p=0.0316
p=0.0417
h)
naive CD8+ T cells [%]
p=0.0092
i)
effector CD8+ T cells [%]
p=0.0016
p=0.0434
j)
Ly6G+Ly6C+ cells of CD11b+ cells [%]
p<0.0001
p<0.0001
p=0.0074
p=0.0162
k)
Ly6Chigh cells of CD11b+ cells [%]
WT
RK
BL KO
RK-BL KO
BLKO

Suppl.Fig. 6
